# Structure, function, regulation, evolution, and therapeutic implications of PARP14

**DOI:** 10.1101/gad.353495.125

**Published:** 2026-06-01

**Authors:** Pulak Kar, Nina Đukić, Dragana Ahel, Ivan Ahel

**Affiliations:** 1 Department of Biological Sciences, Sri Ramaswamy Memorial University-Andhra Pradesh, Amaravati 522240, India;; 2 Sir William Dunn School of Pathology, University of Oxford, Oxford OX1 3RE, United Kingdom

**Keywords:** PARP, ADP-ribosylation, ubiquitin, interferon signaling, infection, DNA damage

## Abstract

ADP-ribosylation (ADPr) is a posttranslational modification conserved across all domains of life that regulates a wide variety of processes. Among ADP-ribosyl transferases (ARTs) in humans, PARP14 has emerged as a key regulator involved in antiviral defense, inflammation, and DNA damage response. Recent studies have uncovered its dynamic and versatile roles in modulating DNA replication stress and DNA repair, positioning it at the intersection of genomic stability and immune regulation. Despite growing insights into its molecular functions, many aspects of PARP14-mediated signaling remain elusive. In this perspective, we explore the fundamental and emerging biological features of PARP14, highlighting its structure, function, evolution, and roles in cellular homeostasis and disease. Additionally, we discuss recent advances in targeting PARP14 for therapeutic intervention, particularly in oncology and immunology. A deeper mechanistic understanding of PARP14 function may inform the development of novel therapeutic strategies that exploit its regulatory network for clinical benefit.

## Abstract (precis)

In this Perspective, Kar et al. discuss all things PARP14, an ADP-ribosyl transferase that demonstrates notable versatility in its functions, with roles in antiviral response, inflammation, maintaining genome stability, and more. Here, the authors discuss the molecular details of PARP14's structure, function, and evolution and present PARP14 as an interesting therapeutic target or biomarker.

ADP-ribosylation (ADPr) is a modification catalyzed by ADP-ribosyl transferase (ART) enzymes that use nicotinamide adenine dinucleotide (NAD^+^) as a cosubstrate to transfer dinucleotide ADP-ribose tags to different macromolecules (Groslambert et al. 2021; Suskiewicz et al. 2023b). ADPr was discovered six decades ago through work on the vertebrate PARP1 ART enzyme and bacterial toxins, such as diphtheria toxin (Chambon et al. 1963; Oppenheimer and Bodley 1981). Over time, many new ART enzymes have been uncovered, represented in organisms across all evolutionary branches (Perina et al. 2014). Humans possess the largest ART family, consisting of 17 PARP proteins that intricately regulate essential functions such as DNA repair, immunity, apoptosis, behavior, and development (Beck et al. 2014; Mikolčević et al. 2021; Lüscher et al. 2022; Biaesch et al. 2023; Suskiewicz et al. 2023b). Some PARP enzymes, including the best-characterized member, PARP1, synthesize chains of repeating ADP-ribose units on proteins, a modification referred to as poly-ADP-ribosylation (PARylation) (Langelier et al. 2012; Palazzo and Ahel 2018; Huang and Kraus 2022). In contrast, most other PARPs catalyze mono-ADP-ribosylation (MARylation), adding only a single ADP-ribose (Vyas et al. 2014).

ADPr can be attached to both proteins and nucleic acids (Groslambert et al. 2021). In proteins, ADPr can modify different amino acid side chains, depending on the specificity of the ADP-ribosylation system involved. For example, in DNA damage response, the main targets are serine residues, and these modifications are synthesized upon detection of DNA breaks by the action of PARP1/PARP2 in the obligatory complex with HPF1 (Palazzo et al. 2018; Suskiewicz et al. 2020). Many PARP family members, including PARP1 and PARP14, target acidic residues (glutamate and aspartate) (Huang et al. 2020; Đukić et al. 2023). Some PARPs, such as PARP1, PARP10, and PARP14, can modify nucleic acids either on nucleotide bases or on terminal phosphate groups at the ends/breaks; however, the physiological relevance of these reactions remains unclear (Talhaoui et al. 2016; Munnur et al. 2019; Musheev et al. 2022; Suskiewicz et al. 2023b; Wondisford et al. 2024).

Like most posttranslational modifications, ADPr is a reversible process. The reversal is mediated by three families of hydrolytic protein domains: macrodomains, ARH domains, and NADAR domains (Rack et al. 2020a; Schuller et al. 2023). As is the case with the transferase enzymes, ADP-ribosyl hydrolases have different specificities: Serine-linked ADPr is reversed by the ARH3 enzyme (Fontana et al. 2017), while glutamate-linked ADPr is targeted by TARG1, MACROD1, and MACROD2 proteins (Jankevicius et al. 2013; Rosenthal et al. 2013; Sharifi et al. 2013). These three hydrolases also remove the ADPr from the phosphate groups at the ends of nucleic acids (Munnur and Ahel 2017; Munnur et al. 2019).

ADPr signals are read by different reader proteins to control a variety of cellular pathways (Teloni and Altmeyer 2016). This perspective focuses on the roles of ADPr in immunity, inflammation, and DNA damage response, with a particular focus on PARP14, which operates at the intersection of these processes.

## Interferon-induced PARPs

While PARP1/2-mediated ADPr and its roles in DNA repair are well characterized (Eisemann and Pascal 2020; Palazzo et al. 2021), our understanding of ADPr by other human PARPs remains comparatively limited. Within the poorly understood subcategories lies the group of interferon (IFN)-induced “antiviral PARPs” including PARP7, PARP9, PARP10, PARP11, PARP12, PARP13, and PARP14. These PARP proteins possess a set of putative RNA binding domains and a catalytic PARP-type ART domain at the C terminus and act as MARylating rather than PARylating enzymes (Suskiewicz et al. 2023a). Positive evolutionary selection in these PARPs suggests a coevolutionary response to viral challenges, indicating significant host–virus interplay (Kerns et al. 2008; Daugherty et al. 2014; Gossmann and Ziegler 2014). Many of these PARPs also have a broader impact on immunity processes and inflammation (Brady et al. 2019; Santinelli-Pestana et al. 2023).

Several members of the antiviral PARP subfamily play diverse antiviral roles through catalytic and noncatalytic mechanisms (Fehr et al. 2020; Parthasarathy et al. 2025). For example, PARP12 restricts RNA viruses such as Zika virus (ZIKV) and Chikungunya virus (CHIKV) by ADP-ribosylating viral proteins (e.g., NS1 and NS3) and promoting their proteasomal degradation. During infection, PARP12 often localizes to stress granules (Atasheva et al. 2012; Li et al. 2018, 2021). On the other hand, PARP10, which is active against alphaviruses such as Sindbis virus (SINV) and CHIKV, mediates mono-ADP-ribosylation of both host and viral (e.g., CHIKV nsP2 protease) targets (Krieg et al. 2023). PARP11 is a further example, playing a dual role in antiviral defense, where it can directly inhibit viral replication and modulate type I interferon (IFN-I) signaling during vesicular stomatitis virus (VSV) or Sendai virus (SeV) infection. Mechanistically, PARP11 mono-ADP-ribosylates the E3 ligase β-TrCP and thereby promotes degradation of the interferon receptor subunit IFNAR1 (Guo et al. 2019). PARP11 also restricts Zika virus replication independently of its enzymatic activity by interacting with PARP12 to promote proteasomal degradation of viral NS1 and NS3 proteins (Li et al. 2021). Unlike catalytically active antiviral PARPs, PARP13 (also known as the zinc finger antiviral protein [ZAP], encoded by the *ZC3HAV1* gene) lacks enzymatic activity. Instead, it recognizes CpG-rich viral RNAs through its CCCH zinc finger domains (Meagher et al. 2019). PARP13 then recruits RNA decay complexes, including TRIM25, KHNYN, and the exosome, to promote degradation of viral transcripts. Through this mechanism, it effectively restricts viruses such as HIV-1, alphaviruses, and SARS-CoV-2 (Guo et al. 2004; Ficarelli et al. 2019; Meagher et al. 2019). Moreover, PARP13 plays a critical role in innate antiviral immunity by enhancing retinoic acid-inducible gene I (RIG-I)-mediated signaling. Specifically, upon detection of the 5′-triphosphate RNA, a hallmark of viral infection, PARP13 directly interacts with RIG-I, promoting its oligomerization and ATPase activity. This enhances downstream activation of IRF3 and nuclear factor κB (NF-κB), leading to robust production of IFN-I and proinflammatory cytokines (Hayakawa et al. 2011). Notably, loss of PARP13 significantly impairs IFN-α/β induction during viral infection, underscoring its essential role in antiviral defense (Hayakawa et al. 2011). These foundational studies demonstrate that PARP13 restricts a variety of RNA viruses, including retroviruses, alphaviruses, and coronaviruses (e.g., SARS-CoV), by binding specific viral RNAs and promoting their degradation, thus functioning independently of its ADP-ribosyl transferase (PARP) catalytic activity (Gao et al. 2002; Guo et al. 2004). In contrast, PARP7, although induced by type I interferons, has been shown to play a potentially proviral role in certain contexts (Grunewald et al. 2020).

## PARP14

**Figure 1.**
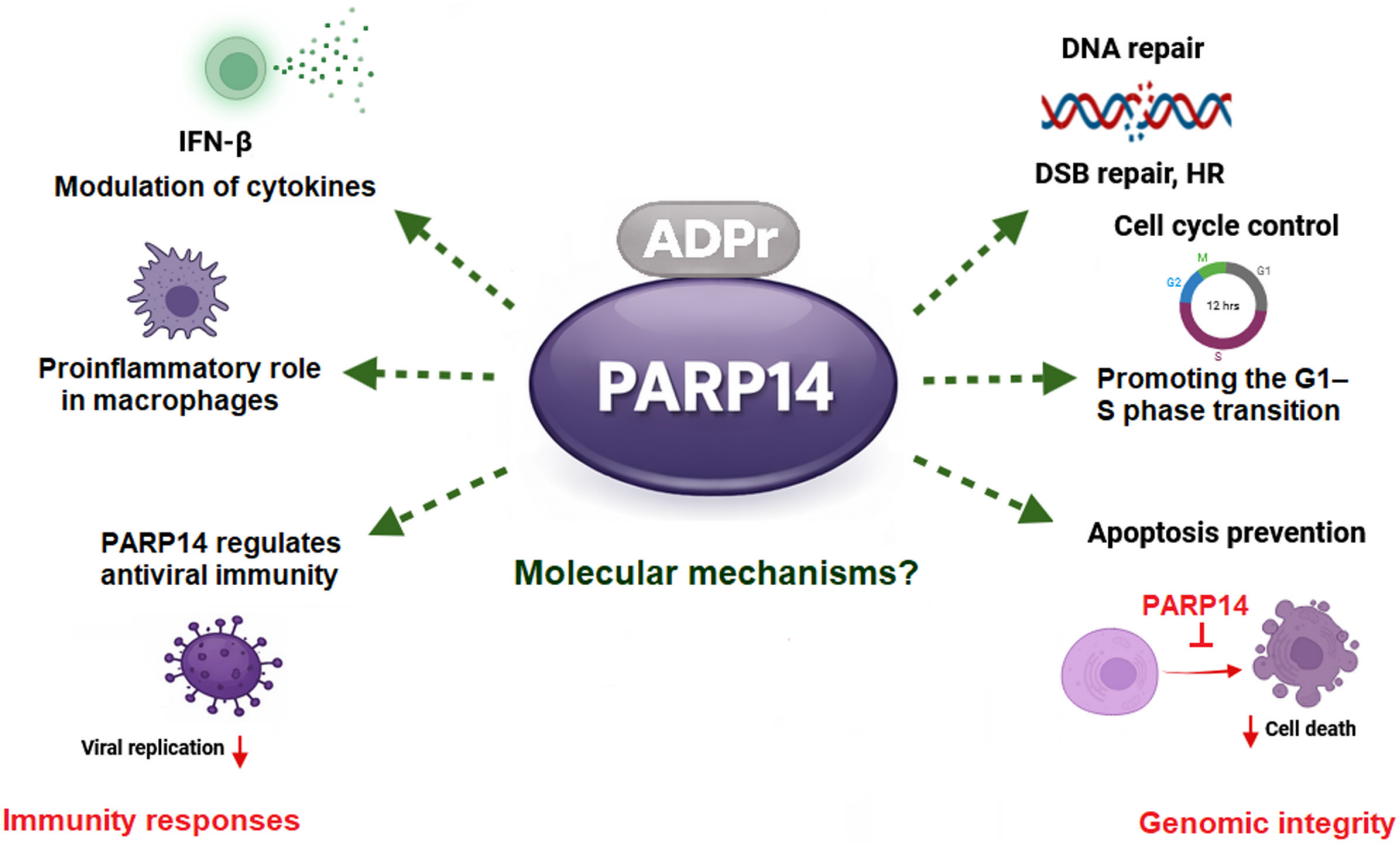
Schematic overview of the roles of PARP14 in immunity and genome stability.

Within the group of antiviral PARPs, PARP14 emerges as a very interesting and versatile player. PARP14 is best known as the main antiviral PARP that suppresses coronavirus infection (Grunewald et al. 2019; Parthasarathy et al. 2025). However, its functions extend beyond antiviral defense to many other important processes such as inflammation, cancer immunity, and DNA damage response (Fig. 1; Schenkel et al. 2021; Dhoonmoon et al. 2022; Mashimo et al. 2022; Santinelli-Pestana et al. 2023; Wong et al. 2023). PARP14 is genetically, evolutionarily, and functionally closely linked to PARP9 (another interferon-induced PARP) and its binding partner, the E3 ubiquitin ligase DTX3L (discussed further below).

## Structure and function of PARP14 domains

**Figure 2.**
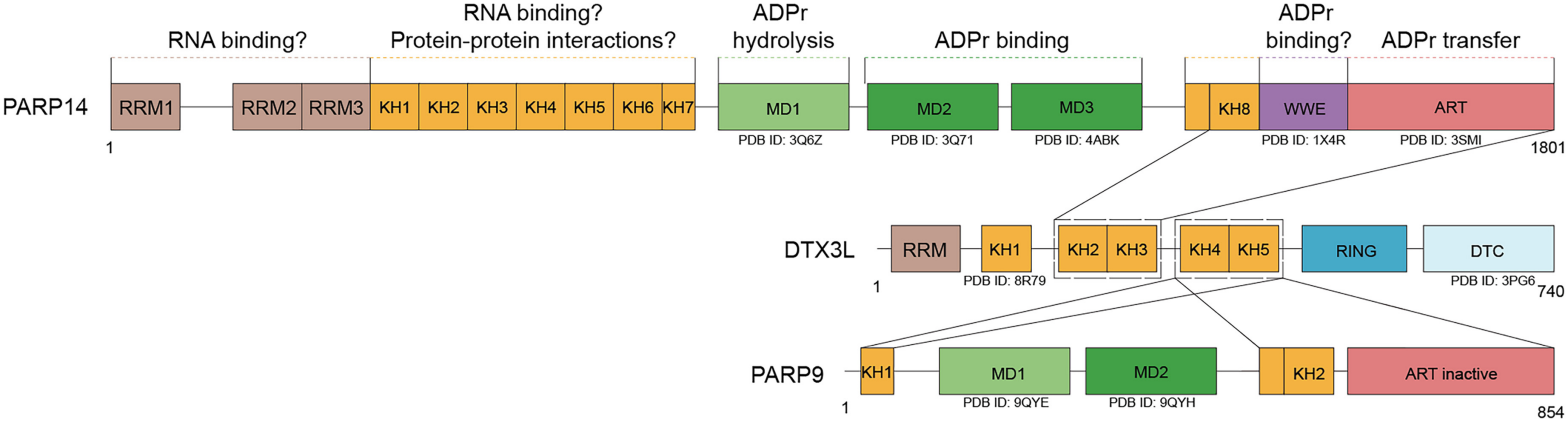
Domain organization and predicted interactions among PARP14, DTX3L, and PARP9. Schematic representation of the domain structures of PARP14, DTX3L, and PARP9. PARP14 contains three RNA recognition motifs (RRM1–3), eight K homology domains (KH1–8) potentially involved in RNA binding and/or protein–protein interactions, three macrodomains (MD1–3) mediating ADP-ribosylation (ADPr) hydrolysis (MD1) and binding (MD2 and MD3), a WWE domain that is a putative ADPr binding module, and a catalytically active ADP-ribosyl transferase (ART) domain. DTX3L comprises an N-terminal RRM, five KH domains (KH1–5), a RING domain, and a DTC domain. PARP9 contains two KH domains (KH1 and KH2); two macrodomains (MD1 and MD2), of which MD1 is hydrolytically active; and an inactive ART domain. Experimentally determined structures of individual domains are indicated by their corresponding PDB IDs. These structures represent isolated domains and are shown for reference. Predicted domain–domain contacts between proteins are based on AlphaFold and marked with black connectors: DTX3L KH2–KH3 interact with the KH8–WWE–ART region of PARP14, while DTX3L KH4–KH5 interact with the KH1–KH2–ART region of PARP9. The tandem KH2–KH5 segment of DTX3L, which forms the interaction core, is outlined by dashed boxes.

As the largest human PARP, PARP14 consists of 1801 amino acids and is composed of a number of distinct protein domains, most of which have not been characterized or have been poorly characterized (Fig. 2). The apo structures are available for several isolated domains of PARP14 (Fig. 2; Forst et al. 2013). Elucidating the exact biochemical activities and specificities of these domains will be crucial for understanding the physiological functions and regulation of PARP14 (Dhoonmoon and Nicolae 2023; Suskiewicz et al. 2023b).

The ART activity of PARP14 is limited to mono-ADP-ribosylation due to a leucine residue that occupies the position of the catalytic glutamate required for polymer formation (Kleine et al. 2008). PARP14 was shown to predominantly modify aspartate and glutamate residues (Carter-O'Connell et al. 2018; Rack et al. 2020b; Wallace et al. 2021; Đukić et al. 2023). PARP14 ADPr activity is significantly enhanced upon activation of interferon signaling (Kar et al. 2024; Ribeiro et al. 2024). Although efforts are ongoing to define its physiological substrates (Carter-O'Connell et al. 2018; Đukić et al. 2023), biochemical and cellular data support robust auto-mono-ADP-ribosylation of PARP14 (Carter-O'Connell et al. 2018; Rack et al. 2020b; Đukić et al. 2023; Javed et al. 2023; Tashiro et al. 2023). The exact modification sites and physiological consequences of these auto-ADPr events are not well understood; however, it has been shown that ADPr of PARP14 facilitates the formation of large cytoplasmic foci containing numerous copies of this protein (Đukić et al. 2023; Ribeiro et al. 2025). Moreover, auto-ADP-ribosylation of PARP14 may modulate its abundance, potentially by altering stability and turnover, though the mechanism remains unclear (Kar et al. 2024). Intriguingly, recent in vitro studies indicate that PARP14 can also ADP-ribosylate nucleic acids (including phosphate groups at the ends or breaks of RNA or DNA) (Đukić et al. 2023; Suskiewicz et al. 2023a), raising the possibility that PARP14 might exhibit dual cellular activity on both protein and nucleic acid substrates. This dual functionality hints at broader regulatory roles for PARP14 in cellular stress signaling and RNA metabolism, which remain important areas for future investigation.

In addition to the ART domain, PARP14 features three macrodomains (MD1, MD2, and MD3) (Fig. 2). Macrodomains are evolutionarily widespread ADP-ribose-binding modules that are present in some ADPr hydrolases. When catalytically inactive, they can serve as reader domains that interpret ADP-ribosylation to modulate downstream cellular signaling (Rack et al. 2016). All PARP14 macrodomains possess ADP-ribose-binding capability; however, MD1 exhibits relatively weak binding activity (Forst et al. 2013; Gibson et al. 2017). Recently, it was discovered that the MD1 of PARP14 and the equivalent macrodomain in PARP9 exhibit glycohydrolase activity, which removes ADP-ribose from cellular targets, and this finding mechanistically explains their low ADP-ribose binding capacity (Fig. 2; Delgado-Rodriguez et al. 2023; Đukić et al. 2023; Torretta et al. 2023). These macrodomains are efficient in reversing Glu–ADPr and Asp–ADPr on automodified PARP14 and many other cellular proteins (Đukić et al. 2023; Kar et al. 2024). Self-standing human ADPr hydrolases such as MACROD1 and TARG1 may also be able to hydrolyze PARP14-mediated modification (Đukić et al. 2023; Challa et al. 2025). Notably, these hydrolases along with PARP14 and PARP9 MD1 can also hydrolyze the phosphate-linked ADPr at the ends or breaks of nucleic acid substrates (Munnur and Ahel 2017). Furthermore, the same activities on protein and nucleic acid substrates are shared by the homologous macrodomains in a number of viruses, including the Mac1 macrodomain module in the Nsp3 protein of coronaviruses (Fehr et al. 2016, 2018; Li et al. 2016; Abraham et al. 2018; Munnur et al. 2019; Rack et al. 2020b; Đukić et al. 2023). Taken altogether, PARP14 is the only PARP with both transferase and hydrolase activities. It is predicted that PARP14 uses these antagonistic functions to fine-tune its own ADPr activity (Đukić et al. 2023). Physiological targets of the PARP14 MD2 and MD3 reader domain need to be established, but in an overexpression system, they were shown to bind mono-ADP-ribosylated PARP10 protein (Forst et al. 2013).

The N-terminal region of PARP14 comprises a series of domains predicted to bind nucleic acids: three RNA recognition motifs (RRMs) and eight K homology (KH) domains. RRM and KH domains are among the most evolutionarily widespread RNA-binding modules, but in PARP14, they largely have not been characterized. In other proteins, the RRM domains usually recognize nucleotide sequences of ∼4–6 bases in a sequence-independent manner (Maris et al. 2005). KH domains usually recognize RNA or DNA in a sequence-dependent manner (Valverde et al. 2008), and it was suggested that at least some KH domains of PARP14 possess DNA or RNA binding capability (Suskiewicz et al. 2023a; Zhu et al. 2024a). In some proteins, such as human poly(C)-binding protein (PCBP), KH domains can mediate protein–protein interactions (Du et al. 2008). In PARP14, the last (eighth) KH domain was shown to directly interact with the second and third KH domains of its binding partner, the E3 ubiquitin ligase DTX3L (Fig. 2; Kar et al. 2024; Saleh et al. 2024). The interactions between PARP14 and DTX3L are discussed in further detail below. Together, these findings suggest that the RRM and KH domains of PARP14 may participate in nucleic acid binding and, in the case of the KH domains, also in mediating protein–protein interactions; however, their precise physiological roles remain to be determined.

A WWE domain precedes the catalytic ART domain in the C-terminal end of PARP14. The WWE domains contain a conserved Trp–Trp–Glu motif and are typically involved in binding PAR chains (Wahlberg et al. 2012). Such activity, however, was not detected for the PARP14 WWE domain (Kleine et al. 2008), and its function remains unknown.

## PARP14 interactors and ADP-ribosylation targets

The size of PARP14 and its broad functionality suggest interactions with diverse proteins and the modification of numerous substrates via ADPr. PARP14 forms complexes with DTX3L and PARP9 (Takeyama et al. 2003; Bachmann et al. 2014; Caprara et al. 2018; Ribeiro et al. 2024) and is tightly linked to ubiquitination (this will be explained in more detail below). PARP14 directly interacts with phosphoglucose isomerase (PGI), stabilizing it by suppressing ubiquitination and promoting its secretion, thereby potentially enhancing tumor cell motility and metastatic behavior (Yanagawa et al. 2007). PARP14 was further shown to interact with and modify autophagy receptor p62/SQSTM1 (Caprara et al. 2018; Đukić et al. 2023; Raja et al. 2025). Upon induction of PARP14 activity through either immune stimulation or overexpression of PARP14, PARP14 localizes to ADP-ribosylated condensates in the cytoplasm (Đukić et al. 2023). These condensates show colocalization with a subset of p62 bodies (Raja et al. 2025). The formation of these p62 bodies depends on the ubiquitin–proteasome system and is not affected by autophagy inhibition. Similarly to p62, PARP14 both binds and modifies histone deacetylase 2 (HDAC2), which was suggested to serve as a transcriptional switch for STAT6-dependent gene induction (Mehrotra et al. 2011). Another notable target for PARP14 ADP-ribosylation is STAT1, which modulates inflammation (Iwata et al. 2016). Finally, PARP14 interacts with DDX6, a helicase critical for storing mRNA within P-bodies (Carter-O'Connell et al. 2018; Đukić et al. 2023). The physiological consequences of PARP14 association with all these proteins are not well understood.

Many of the modifications by PARP14 are fine-tuned through reversal by the hydrolytic activity of its first macrodomain (MD1). These include modifications of immune proteins (PARP13, RNF114, and RNF166) and DNA damage response (DDR) factors (PARP1, XRCC1, and RPA1), as well as the tankyrase PARPs and their substrates, such as AMOTL1 and ZNRD2/SSSCA1 (Đukić et al. 2023).

Collectively, these findings emphasize PARP14's dual role as a structural scaffold and as an enzymatic regulator by reversible ADPr. However, despite identification of many substrates, the precise ADPr modification sites on most targets remain unresolved due to mass spectrometry limitations in detecting modifications on glutamate and aspartate residues—with the exception of PARP14 automodification site at aspartate 1604 and ADPr sites on PARP13, which have been mapped with higher confidence (Carter-O'Connell et al. 2018; Higashi et al. 2019). Recent technical breakthroughs in the detection of ADPr sites on acidic residues revealed a number of additional modification sites on PARP14 in cells upon interferon stimulation (Buch-Larsen et al. 2025). Collectively, these findings highlight that PARP14 integrates protein–protein interactions, catalytic activity, and regulated turnover to adjust immune and stress signaling. Looking ahead, mapping the precise ADPr modification sites on its substrates, understanding the dynamic regulation of PARP14-containing condensates, and exploring its cooperation with the ubiquitin–proteasome system will be important to understanding its physiological function and mechanisms. Such insights could open new therapeutic avenues, where selective modulation of PARP14's scaffolding functions or catalytic activity may provide opportunities to target cancer, inflammation, and infection in a context-specific manner.

## Cross-talk of PARP14 and ubiquitination

Recent studies have revealed a tightly coordinated interplay between PARP14, PARP9, and DTX3L proteins, which are coexpressed from the same genetic locus (at chromosome 3q21.1) (Bachmann et al. 2014; Iwata et al. 2016; Caprara et al. 2018). PARP9 is an ADP-ribosylation-inactive PARP (Vyas et al. 2014) that arose from a relatively recent duplication of the ancestral PARP14 gene (Daugherty et al. 2014). DTX3L is an E3 ubiquitin ligase of the Deltex family (Jiang et al. 2025). Interestingly, PARP9 and DTX3L combined have a domain structure similar to that of PARP14, involving RRM domains, KH domains, macrodomains, and ART domains, apart from additional ubiquitination modules at the C terminus of DTX3L (Fig. 2; Kar et al. 2024; Zhu et al. 2024a).

PARP9 and DTX3L are expressed from a shared bidirectional promoter (Juszczynski et al. 2006) and form a tight complex (Ashok et al. 2022). PARP14 interacts more loosely with the PARP9/DTX3L complex via direct interaction with DTX3L (Kar et al. 2024; Saleh et al. 2024). Specifically, DTX3L has five KH domains similar to the ones found in PARP14 (Fig. 2), and two of these domains (KH2 and KH3) interact with the C-terminal KH8–WWE–ART fragment of PARP14 (Kar et al. 2024). Upon interferon stimulation, these three proteins colocalize in numerous copies at specific cytosolic foci (Kar et al. 2024; Ribeiro et al. 2024). However, their interplay at these sites is complex and not yet well understood. DTX3L seems to exhibit both positive and negative effects on PARP14. On the one hand, it is required for stabilization of PARP14 protein levels upon induction by interferon through at least partly proteasome-dependent posttranslational regulation (Kar et al. 2024; Ribeiro et al. 2024). DTX3L is also required for the localization of PARP14 and interferon-induced ADPr at the cytoplasmic foci (Kar et al. 2024). On the other hand, DTX3L restricts PARP14 ADPr activity on selected proteins by physical interaction, which leads to striking changes in the patterns of protein ADP-ribosylation profiles as judged by Western blotting (Kar et al. 2024). In turn, PARP14 is predicted to regulate DTX3L. DTX3L was shown to be a direct target for ADPr by PARP14 (Kar et al. 2024; Ribeiro et al. 2024). When both proteins are expressed, PARP14 and DTX3L produce ADPr and ubiquitination signals that colocalize in the cytoplasm (Kar et al. 2024). It is not clear what these modification events actually target or their physiological relevance, but they seem to be negatively regulated by the hydrolytic function of PARP9 MD1 (Kar et al. 2024). It is plausible that some of these colocalizing ADPr and ubiquitination signals represent the recently discovered dual, hybrid modification referred to as ADPr–Ub or MARubylation (Zhu et al. 2022a, 2024a; Bejan et al. 2025; Chatrin et al. 2025). The PARP14/DTX3L-mediated ADPr–Ub signals may be extended by E3 ubiquitin ligases RNF114 and RNF166 to include ubiquitin chains (Kloet et al. 2025; Lacoursiere et al. 2025). Altogether, current insights suggest the striking complexity of the interplay between ADPr and ubiquitination signaling in this system. The underlying mechanisms and physiological implications will hopefully be clarified by future studies (Zhu et al. 2025).

## Evolution of PARP14

PARP14 belongs to clade 3 PARPs along with other interferon-inducible PARPs, such as PARP7, PARP10, and PARP12 (Perina et al. 2014). This clade is evolutionarily distinct from the better understood DNA repair PARPs or tankyrase PARPs (clades 1 and 4, respectively) (Palazzo and Ahel 2018). Coevolution with viruses possibly led to the fast evolution of antiviral PARPs, indicating their role in host–pathogen conflicts (Delgado-Rodriguez et al. 2023). Among antiviral PARPs, PARP14 shows one of the highest evolutionary rates, particularly within its macrodomains and ART domain, which display clear signatures of positive selection (Gossmann and Schmid 2011; Gossmann and Ziegler 2014). Several positively selected sites are located near domain interaction interfaces, especially between macrodomains 1 and 2, indicating potential coevolutionary adaptations that may influence substrate recognition rather than NAD^+^ binding (Gossmann and Ziegler 2014). These findings indicate functional diversification in substrate interaction sites of NAD^+^-consuming enzymes, positioning PARP14 as an example of evolutionary plasticity among ADP-ribosyl transferases.

PARP9 and PARP15 are the human PARPs most closely related to PARP14. They are believed to have arisen from partial duplications of the PARP14 gene in vertebrates and mammals, respectively. This evolutionary divergence likely enhanced signaling efficiency and broadened the antiviral host repertoire (Perina et al. 2014). Both PARP9 and PARP15 contain two macrodomains that are N-terminal to the ART domain (Suskiewicz et al. 2023a). As mentioned above, PARP9 is suggested as a direct regulator of PARP14 (Kar et al. 2024), while PARP15 is a poorly understood catalytically active PARP with a domain structure similar to those of PARP14 and PARP9 (Biaesch et al. 2023; Suskiewicz et al. 2023a; Ebenwaldner et al. 2025). It was suggested that PARP14 arose 700 million years ago, followed by PARP9 (∼500 million years) and PARP15 (∼100 million years) (Kumar and Hedges 1998; Delgado-Rodriguez et al. 2023). Duplications of PARP14-like genes often occur close together on chromosomes, as seen in the closest relative of vertebrates, the lancelet *Branchiostoma floridae*, where four PARP14-like proteins cluster in the same genomic location (Perina et al. 2014). Multiple PARP14 paralogs are also found in lower metazoan organisms, including sponges (Perina et al. 2014). This observation supports the idea that PARP14 duplication occurred in response to the diverse range of viruses to which these organisms are exposed.

Viral macrodomain proteins play crucial roles in host–virus conflict by modulating ADP-ribosylation signaling, and their evolution is linked to macrodomains in PARP14 and PARP9 (Daugherty et al. 2014; Grunewald et al. 2019; Đukić et al. 2023). In positive-sense single-stranded RNA viruses like alphaviruses, herpesviruses, and coronaviruses, these macrodomains are essential for replication, virulence, and evasion of the IFN response, highlighting their central role in the battle between host immunity and viral evasion (Li et al. 2016; Fehr et al. 2018; Rack et al. 2020b; Leung et al. 2022; Parthasarathy et al. 2025).

## Roles of PARP14 in infection

**Figure 3.**
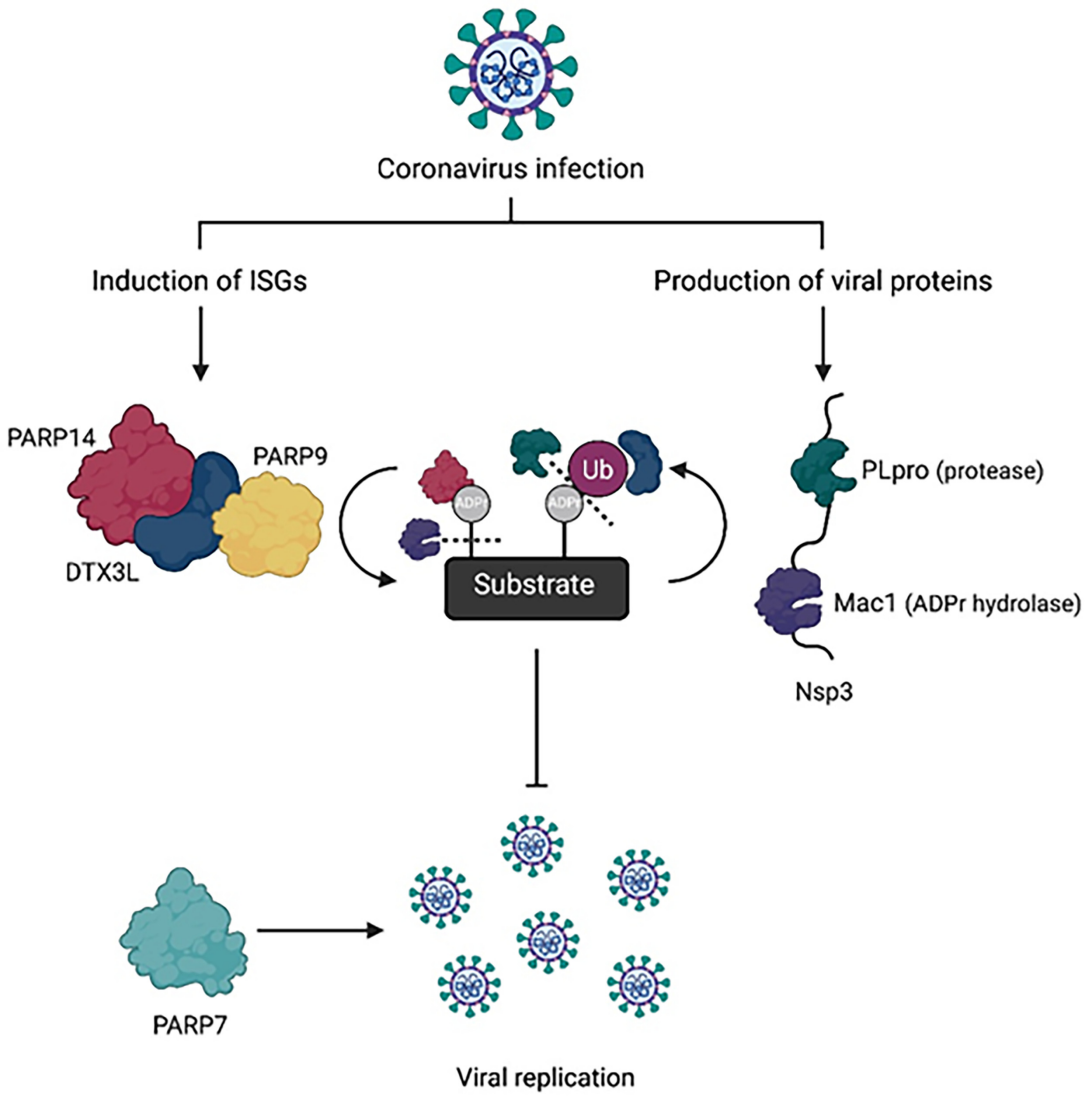
Model of the interplay between host ADP-ribosylation and ubiquitination factors and viral evasion mechanisms during coronavirus infection. The PARP14/DTX3L/PARP9 complex is induced as part of the interferon-stimulated antiviral response, whereas PARP7 has been suggested to promote viral replication. Upon viral infection, PARP14 modifies various host and viral proteins, some of which could undergo dual, hybrid ADP-ribose–ubiquitin (ADPr–Ub) modification mediated by DTX3L. This process might contribute to restricting viral replication, though the specific substrates and underlying molecular mechanisms remain to be defined. Meanwhile, coronaviruses encode proteins such as PLpro (papain-like protease) and Mac1 (an ADP-ribose hydrolase) within nonstructural protein 3 (Nsp3), which can counteract PARP-mediated antiviral activity by reversing ADP-ribosylation and ubiquitination on target substrates.

Fast evolution of PARP14 and its numerous duplications in many organisms clearly support its core function as an antiviral enzyme that aids in adaptation to a wide diversity of pathogens (Daugherty et al. 2014; Gossmann and Ziegler 2014; Perina et al. 2014). PARP14 is a focal point in ADPr research, particularly given its role as the main antiviral PARP that suppresses coronavirus infections (Grunewald et al. 2019; Parthasarathy et al. 2025). Initial evidence using the mouse coronavirus (MHV) model showed that depleting or inhibiting PARP14 increased replication of the viral Mac1 catalytic mutant (Mac1 ADPr hydrolase-deficient Nsp3 protein of the MHV), with little to no effect on the WT virus (Grunewald et al. 2019). With improved tools (PARP14 KO cells, a PARP14-deficient mouse, and a potent, selective PARP14 inhibitor) a recent study confirmed that PARP14 also restricts infection by human SARS-CoV-2 (Parthasarathy et al. 2025). These findings support a model in which viral macrodomains (e.g., Mac1) antagonize PARP14-mediated antiviral ADP-ribosylation (Fig. 3).

More recently, the role of PARP14 was expanded to a wider range of viruses (Parthasarathy et al. 2025). In addition to SARS-CoV-2, PARP14 expression also rises during infections with other viruses like Chikungunya virus, Zika virus, and β-coronaviruses (Parthasarathy and Fehr 2022). PARP14 limited the replication of the double-stranded DNA virus herpes simplex virus type 1 (HSV-1) but increased the replication of the negative-sense RNA virus vesicular stomatitis virus (VSV). Interestingly, both effects were independent of its ADPr activity, and the VSV phenotype occurred without detectable changes in interferon production (Parthasarathy et al. 2025), suggesting potential roles of other PARP14 domains, such as RNA-binding domains or macrodomains, in regulating the viral infection response.

How PARP14 restrains viruses is not fully understood, but one potential mechanism involves the direct ADPr-mediated targeting of viral proteins or viral nucleic acids to inactivate them. An alternative mechanism may involve promotion of the interferon response through modification of specific host proteins. An example of the latter mechanism was demonstrated in the response to coronavirus infection (Grunewald et al. 2019; Parthasarathy et al. 2025). For instance, during infection with Mac1 mutant MHV and Mac1-deficient SARS-CoV-2, PARP14 ADPr activity promoted IFN-β production (Grunewald et al. 2019; Parthasarathy et al. 2025). In support of this, PARP14 promotes IFN-β expression in LPS-stimulated macrophages, which is associated with decreased replication of the intracellular bacterium *Salmonella typhimurium* (Caprara et al. 2018). Notably, PARP14 interactor PARP9 was shown to influence *Mycobacterium tuberculosis* infection by enhancing IFN-γ-induced antibacterial pathways and regulating autophagy-associated responses, thereby strengthening host defense mechanisms (Zhu et al. 2023). Although PARP14 in some models enhances IFN-β and IFN-λ production (Caprara et al. 2018; Grunewald et al. 2019; Parthasarathy et al. 2025), its effect appears to be highly context-dependent. For instance, PARP14 inhibition significantly reduced EMCV-induced interferon responses but had no effect on Sendai virus-induced IFN-β, indicating a stimulus-specific regulation of interferon pathways (Parthasarathy et al. 2025).

Viruses readily evolve ways to counteract antiviral proteins. The same is true for the PARP14 system. Genetic evidence clearly shows that the macrodomain protein module Mac1 within the Nsp3 protein of coronaviruses is essential for counteracting PARP14's antiviral activity (Fig. 3; Grunewald et al. 2019). Mac1 has ADP-ribosyl hydrolase activity and is able to reverse PARP14 ADPr (Rack et al. 2020b; Đukić et al. 2023; Kar et al. 2024). As mentioned above, Mac1 is essential for suppressing the antiviral function of PARP14 in vivo (Grunewald et al. 2019). Domains homologous to Mac1 are found in a variety of other viruses, including alphaviruses and the Rubella virus (Li et al. 2016; Leung et al. 2022). Evolutionary analyses support the hypothesis that viral Mac1 and similar domains in other viruses may have arisen via the capture and adaptation of a host PARP14-like MD1 that was subsequently refined by viral selection pressures for immune antagonism (Delgado-Rodriguez et al. 2023; Đukić et al. 2023).

The precise ADPr sites targeted by Mac1 and their physiological consequences remain unknown (Parthasarathy and Fehr 2022), but it is expected that they reverse some modifications mediated by PARP14 (Đukić et al. 2023; Kar et al. 2024), which may be on host or viral proteins or even on viral nucleic acids. As mentioned previously, PARP14 cooperates with the E3 ligase DTX3L to conjugate ubiquitin to ADPr-modified substrates, possibly generating dual ADPr–Ub marks (Kar et al. 2024; Chatrin et al. 2025; Zhu et al. 2025). Notably, the Ub hydrolase domain PLpro, which is adjacent to the Mac1 domain within the Nsp3 protein, was shown to remove these ubiquitination modifications in vitro (Zhu et al. 2022a, 2024a), suggesting that such ADPr-dependent ubiquitination could function as a part of virus–host conflict (Zhu et al. 2022a; Chatrin et al. 2025). Altogether, these studies and the fact that both Mac1 and PLpro are important for viral replication spurred the efforts to develop Mac1 and PLpro inhibitors as potential antiviral drug targets (Schuller et al. 2021b; Roy et al. 2022; Gahbauer et al. 2023; Pfannenstiel et al. 2025; Suryawanshi et al. 2025).

## Roles of PARP14 in inflammation

PARP14 has been implicated in the regulation of inflammation by balancing proinflammatory and anti-inflammatory signals orchestrating both transcriptional and posttranscriptional programs downstream from cytokine pathways (Mehrotra et al. 2011; Iwata et al. 2016; Boehi et al. 2021). In Th2 immunity, PARP14 acts as a STAT6-dependent transcriptional coactivator, displacing HDAC2/3 and recruiting histone acetyltransferases at STAT6-responsive promoters, thereby promoting H3K27 acetylation and RNA polymerase II loading (Mehrotra et al. 2011). This activity enhances Th2 cytokine expression (IL-4, IL-5, and IL-13) and drives allergic airway inflammation while also antagonizing STAT1-mediated proinflammatory transcription to fine-tune Th1/Th2 balance (Mehrotra et al. 2011; Iwata et al. 2016). In some models of inflammation, PARP14 does not directly affect transcription (Schenkel et al. 2021). For instance, PARP14 contributes to mRNA stability by interacting with tristetraprolin (TTP), controlling the decay of inflammatory transcripts such as tissue factor in macrophages (Iqbal et al. 2014). In vivo, PARP14 deficiency leads to reduced Th17/Th2 cytokine production and attenuated allergic airway inflammation (Mehrotra et al. 2015). PARP14 also shapes IFN-γ signaling in macrophages, where it suppresses proinflammatory responses, whereas PARP9 exerts opposing anti-inflammatory effects; together, they fine-tune STAT1 ADP-ribosylation to maintain immune balance (Iwata et al. 2016; Fehr et al. 2020; Boehi et al. 2021). Furthermore, PARP14 regulates type I IFN responses by modulating nuclear accumulation of IFN-inducible proteins (Caprara et al. 2018). Importantly, PARP14 inhibitors display potent anti-inflammatory effects, reversing IL-4-driven protumor gene expression in macrophages (Schenkel et al. 2021) and suppressing cytokine production in a mouse model of atopic dermatitis (Wu et al. 2025).

## Roles of PARP14 in genome stability

Early evidence showed that PARP14 supports genome stability, as the loss of PARP14 protein causes homologous recombination DNA repair defects and DNA replication stress in cellular systems (Fig. 1; Nicolae et al. 2015; Dhoonmoon and Nicolae 2023). A CRISPR screen revealed that the phosphorylation signal cascade induced during the ATR–CHK1-dependent DNA damage response is important for survival of PARP14-deficient cells. These findings underscore PARP14's role in regulating DNA replication dynamics under stress and limiting the amount of DNA damage (Dhoonmoon et al. 2020). DNA repair factors modified by PARP14 are the DNA break sensor KU complex and the MRE11 nuclease (Carter-O'Connell et al. 2018), and it was shown that the KU–PARP14 axis regulates DNA resection at stalled replication forks through MRE11 and EXO1 (Dhoonmoon et al. 2022). While mechanistic details regarding how ADP-ribosylation of MRE11 and Ku is linked to DNA repair remain unknown, it could be predicted that ADPr of Ku and MRE11 functions as a reversible regulatory signal that modulates their DNA binding capability and/or catalytic activity at DNA lesions.

Proteomic studies also found that PARP14 ADP-ribosylates XRCC1, APLF, RPA1, and several other DDR proteins (Đukić et al. 2023), further suggesting the role of PARP14 in DNA repair. Experimentally, using pull-downs, PARP14 has been identified as an interactor of the RPA complex, a key single-stranded DNA-binding factor involved in DNA repair/replication (Maréchal et al. 2014). Recent work demonstrated that when MD1 of PARP14 is mutated to inactivate its ADPr hydrolase activity, there is an enhanced interaction with RPA, suggesting the role of MD1 in the PARP14-mediated DDR (Đukić et al. 2023). It remains to be seen whether putative DNA/RNA binding domains of PARP14 are important for its roles in DNA damage response, as was shown for the well-characterized PARP1 (Langelier et al. 2012).

Interestingly, available data showed that PARP9 and DTX3L also have roles in the DNA damage response. PARP1-mediated PARylation at DNA damage sites recruits the PARP9/DTX3L complex, which is suggested to ubiquitinate histones to facilitate the recruitment of DDR regulators 53BP1 and BRCA1, thereby limiting DNA damage (Yang et al. 2017). The complex also targets the p53 tumor suppressor for degradation, regulating its levels during DDR (Yang et al. 2017; Hafner et al. 2019; Yan et al. 2023). PARP9's presence at the DNA damage sites relies on its MD1 (Đukić et al. 2023).

Notably, in addition to modifying proteins, the PARP14/PARP9/DTX3L complex may also modify nucleic acids during DDR, as suggested by recent in vitro studies using recombinant proteins. As mentioned above, PARP14 has been shown to directly modify single-stranded nucleic acids at their phosphate ends or breaks (Đukić et al. 2023; Suskiewicz et al. 2023a). Similarly, DTX3L can ubiquitinate nucleic acids directly either via ribose groups (Zhu et al. 2024b) or through ADP-ribose marks (Zhu et al. 2024a). It remains to be determined whether such modifications occur in cells and, if so, whether they influence nuclease processing of DNA breaks or serve to recruit effector proteins that recognize ADPr or ubiquitin marks. Addressing these questions will require major advances in appropriate mass spectrometry approaches, as ADPr of nucleic acids has only recently come into focus (Groslambert et al. 2021; Schuller et al. 2021a; Wondisford et al. 2024; Tang et al. 2026). Alternatively, as PARP14, PARP9, and DTX3L are largely cytoplasmic proteins, they may have a function outside of core DNA repair as modulators of innate immune responses by cGAS (cyclic GMP–AMP synthase) and other sensors of nucleic acid fragments arising from repair and replication stress (Erdal et al. 2017).

## Role of PARP14 in other processes

Beyond its roles in the processes mentioned above, PARP14 has emerged as an important modulator of tumor–immune interactions. Chronic IFN-γ exposure has been shown to drive resistance to PD-1 blockade in cancer models by upregulating PARP14, which attenuates STAT1 activation and downstream immune responses. Strikingly, pharmacological inhibition of PARP14 was sufficient to restore checkpoint sensitivity and reinvigorate antitumor immunity (Wong et al. 2023). Complementary translational studies in patients demonstrated that elevated PARP14 expression correlates with poor immunotherapy outcomes, while its inhibition enhances tumor immunogenicity and therapeutic response (Leshem et al. 2025). Together, these findings identify PARP14 as a regulator of adaptive resistance to immune checkpoint blockade, highlighting its potential as a therapeutic target to overcome immunotherapy resistance.

Expanding on this immunomodulatory role, PARP14 functions in concert with PARP9–DTX3L complexes, which have been shown to modulate tumor immunity by shaping interferon signaling and antigen presentation pathways, thereby influencing tumor–immune dynamics (Zhang et al. 2015; Brooks et al. 2023). In parallel, PARP14-driven ADP-ribosylation influences the tumor microenvironment by regulating macrophage polarization and adaptive immune responses (Wong et al. 2023).

In addition to its immune regulatory roles, PARP14 also impacts cell-intrinsic processes. It promotes cell cycle progression by stabilizing cyclin D1 mRNA, thereby facilitating the G1–S transition through retinoblastoma (Rb) and p53/p21 pathways. Loss of PARP14 reduces cyclin D1 expression and induces G1 arrest, which can be partially rescued by cyclin D1 overexpression (O'Connor et al. 2021). PARP14 has also been shown to mono-ADP-ribosylate the ribosomal scaffold protein RACK1 in ovarian cancer cells, a modification that promotes its colocalization within stress granules. This PARP14–RACK1 axis is essential for stress granule assembly and supports tumor cell adaptation to stress, linking ADP-ribosylation to survival pathways in malignancy (Challa et al. 2025).

Recent insights further highlight the breadth of PARP14's functions in cancer biology. The PARP9–PARP13–PARP14 axis has been implicated in influencing colorectal cancer response to radiotherapy (Prokarenkaite et al. 2025). In hormone-driven cancers, bioinformatics and experimental analyses revealed that PARP14-driven glycolysis enhances tamoxifen resistance in estrogen receptor-positive breast cancers (Mo et al. 2025). Collectively, these findings show that PARP14 integrates immune regulation, cell cycle control, stress adaptation, metabolic reprogramming, and therapeutic resistance, establishing it as an important factor in cancer biology.

## PARP14 as a biomarker or drug target in disease therapeutics

Given its diverse roles in immunity, inflammation, and cancer, PARP14 has emerged as both a potential biomarker and a therapeutic target. Inhibitors of the ADP-ribosylation activity of PARP14 are promising therapeutics for conditions such as asthma, inflammatory bowel disease, and other immune-mediated disorders (Mehrotra et al. 2013; Iwata et al. 2016; Eddie et al. 2022; Vedantham et al. 2024). For instance, PARP14 inhibitors are solid candidates for clinical trials (Polasek et al. 2025), as they show strong anti-inflammatory effects in vivo, suppressing cytokine production in a mouse model of atopic dermatitis (Wu et al. 2025). Furthermore, a potent and selective PARP14 inhibitor decreased protumor macrophage gene expression and elicited inflammatory responses in tumor explants (Schenkel et al. 2021). In cancer, PARP14 inhibition has been shown to restore PD-1 immune checkpoint sensitivity in IFN-γ-resistant models, underscoring its translational promise for immunotherapy (Wong et al. 2023).

Given the roles of PARP14 MD1 and PARP9 MD1 in reversing PARP14 ADP-ribosylation (Đukić et al. 2023; Kar et al. 2024) and the role of PARP14 ADPr in promoting IFN signaling (Grunewald et al. 2019; Parthasarathy et al. 2025), MD1 macrodomains may also represent promising drug targets. The high similarity of MD1 and viral macrodomains (Rack et al. 2020b) may provide opportunities for dual-eraser inhibitors that target both the viral macrodomain (e.g., SARS2 Mac1) and PARP14/PARP9 MD1. Such a compound could both stabilize PARP14-dependent MARylation and prevent its viral reversal, thereby strengthening host antiviral defense. On the other hand, a selective MD1 inhibitor would be a valuable research tool. With multiple Mac1 inhibitors already in development (Schuller et al. 2021b; O'Connor et al. 2023; Li and Song 2024; Rijpkema et al. 2024; Correy et al. 2025), the next obvious step is to assess cross-activity against PARP9/14 MD1.

PARP14 expression levels correlate with disease severity in inflammatory disorders, viral infections, and multiple cancers, highlighting its predictive and prognostic value (Iansante et al. 2015; Schenkel et al. 2021; Alhammad et al. 2023; Vedantham et al. 2024; Zhang et al. 2024; Mo et al. 2025; Parthasarathy et al. 2025). In cancer, PARP14 is markedly upregulated compared with adjacent nontumor tissue in glioblastoma, where elevated PARP14 expression correlates with poor patient survival (Zhang et al. 2024). There are additional preclinical data suggesting that PARP14 overexpression is linked to poor prognosis in multiple myeloma, lymphoma, acute myeloid leukemia (AML), and renal carcinoma (Cho et al. 2011; Barbarulo et al. 2013; Schenkel et al. 2021; Zhu et al. 2022b).

## Concluding remarks and future directions

The increasing interest in understanding antiviral PARPs, particularly PARP14, has accelerated the rate of discovery in this field of research in recent years. These initial efforts have already revealed several interesting paradigms and novel activities of PARP14, including its key function in coronavirus suppression, novel biochemical activities (e.g., hydrolase function), and interplay with ubiquitination (Grunewald et al. 2019; Brooks et al. 2023; Đukić et al. 2023; Zhu et al. 2025). These efforts also uncovered a striking complexity of regulation and evolution around PARP14 and its complex with PARP9/DTX3L. PARP14 is linked with a growing number of important processes, but our mechanistic understanding is still poor, limiting potential translational efforts. Nevertheless, the exploration of PARP14 activity in the treatment of inflammatory autoimmune diseases such as atopic dermatitis is very promising, which is underscored by a first-in-human phase 1 trial of selective inhibitors such as RBN012759 and RBN-3143 (Schenkel et al. 2021; Polasek et al. 2025). There is also considerable interest in PARP14 function in terms of modulating cancer immunity by restoring PD-1 immune checkpoint sensitivity or suppressing COVID (Parthasarathy and Fehr 2022; Wong et al. 2023).

One limiting aspect for both fundamental and translational studies is the availability of molecular tools. Selective ART domain PARP14 inhibitors are available; however, we lack the means to target other domains, such as the MD1 and MD2 domains. An early cell-permeable inhibitor of PARP14 MD2 with modest efficacy has been reported (Schüler et al. 2017), but there are currently no inhibitors for the MD1 domains of PARP9 and PARP14. It is worth noting that inhibitors against other interferon-induced PARPs may be of interest for controlling viral infection. PARP7, for example, is a suppressor of interferon response (Gozgit et al. 2021), and it was shown that PARP7 knockdown restricts coronavirus replication (Fig. 3; Grunewald et al. 2020). Therefore, the catalytic ADP-ribosylation PARP7 inhibitors may be an attractive direction for new antivirals. Several efficient, cell-permeable PARP7 inhibitors are already available (Gozgit et al. 2021; Sanderson et al. 2023).

Additionally, production of full-length recombinant PARP14, PARP9, and DTX3L proteins for biochemical and structural studies is challenging, but current efforts have yielded promising constructs for the expression from insect cells or by adding affinity tags that increase protein solubility (Chatzicharalampous and Schüler 2024; Kar et al. 2024). Glu/Asp-linked ADPr is highly labile and short-lived in cells, and another major limitation is the current challenge in developing mass spectrometry-based technologies to map PARP14-specific ADP-ribosylation sites. This makes precise site identification technically demanding and often incomplete. However, novel strategies have recently been proposed that yielded the first potential PARP14 modification sites in cells (Buch-Larsen et al. 2025). Notably, identification of precise modification sites has been crucial for resolving the cellular functions and underlying molecular mechanisms of the DNA repair PARPs: PARP1 and PARP2 (Bonfiglio et al. 2017; Hendriks et al. 2021; Prokhorova et al. 2021).

One fundamental aspect deserving clarification is whether PARP14 modifies nucleic acids in cells (Suskiewicz et al. 2023a; Zhu et al. 2024a) and whether ADPr–ubiquitin cross-talk is physiologically relevant for PARP14 (Kar et al. 2024; Bejan et al. 2025; Kloet et al. 2025; Perrard et al. 2025; Wierbiłowicz et al. 2025; Zhu et al. 2025). Lessons from PARP14/PARP9/DTX3L systems will likely be useful for other PARPs that regulate protein stability through the interplay between ADPr and ubiquitination, such as PARP7, PARP10, or tankyrases (Chatrin et al. 2025; Gorelik et al. 2025; Lacoursiere et al. 2025; Perrard et al. 2025; Wierbiłowicz et al. 2025).

Equally important will be to define the structural determinants that guide PARP14 substrate selection and to determine how its catalytic and scaffolding functions are balanced in different cellular contexts. Finally, resolving whether PARP14 activity integrates with innate immune-sensing pathways (e.g., cGAS–STING) or chromatin remodeling complexes will be key for understanding its full physiological significance (Suskiewicz et al. 2023b; Wang et al. 2025). Addressing these challenges will clarify the physiological and pathological roles of PARP14 and unlock opportunities to exploit it as a biomarker and therapeutic target across inflammation, infection, and cancer. With rapidly advancing technologies and emerging small molecule inhibitors, the field is now poised to move from fundamental discoveries toward translational applications, making PARP14 one of the most intriguing and clinically relevant PARPs to watch in the years ahead.

## Glossary

ADPr: ADP-ribosylation

AMOTL1: angiomotin-like 1

APLF: aprataxin and PNK-like factor

ARH3: ADP-ribosyl hydrolase 3

ART: ADP-ribosyl transferase

ATR: ataxia telangiectasia and rad3-related protein

CCCH: Cys–Cys–Cys–His

cGAS: cyclic GMP–AMP synthase

CHIKV: Chikungunya virus

CHK1: checkpoint kinase 1

DDR: DNA damage response

DTX3L: Deltex E3 ubiquitin ligase 3 like

EXO1: exonuclease 1

GATA3: GATA binding protein 3

H3K27: histone H3 lysine 27

HDAC: histone deacetylase

HPF1: histone PARylation factor 1

IFN-β: interferon-β

IFN-γ: interferon-γ

IFN-I: type I interferon

IL: interleukin

IRF3: interferon regulatory factor 3

KHNYN: KH and NYN domain-containing protein

MACROD1: macrodomain-containing protein 1

MACROD2: macrodomain-containing protein 2

MRE11: meiotic recombination 11 homolog

NAD^+^: nicotinamide adenine dinucleotide

NF-κB: nuclear factor κB

NS1: nonstructural protein 1

NS3: nonstructural protein 3

nsP2: nonstructural protein 2

PARP: poly(ADP-ribose) polymerase

PD-1: programmed cell death protein 1

RIG-I: retinoic acid-inducible gene I

RNF114: Ring finger protein 114

RNF166: Ring finger protein 166

RPA: replication protein A

SARS-CoV-2: severe acute respiratory syndrome coronavirus 2

SINV: Sindbis virus

SSSCA1: Sjogren syndrome/scleroderma autoantigen 1

STAT1: signal transducer and activator of transcription 1

STAT6: signal transducer and activator of transcription 6

STING: stimulator of interferon genes

TARG1: terminal ADP-ribose glycol hydrolase 1

TRIM25: tripartite motif-containing protein 25

XRCC1: X-ray repair cross-complementing protein 1

ZIKV: Zika virus

ZNRD2: zinc ribbon domain-containing protein 2

## References

[GAD353495KARC1] Abraham R, Hauer D, McPherson RL, Utt A, Kirby IT, Cohen MS, Merits A, Leung AKL, Griffin DE. 2018. ADP-ribosyl-binding and hydrolase activities of the alphavirus nsP3 macrodomain are critical for initiation of virus replication. Proc Natl Acad Sci 115: E10457–E10466. 10.1073/pnas.1812130115 30322911 PMC6217424

[GAD353495KARC2] Alhammad YM, Parthasarathy S, Ghimire R, Kerr CM, O'Connor JJ, Pfannenstiel JJ, Chanda D, Miller CA, Baumlin N, Salathe M, 2023. SARS-CoV-2 Mac1 is required for IFN antagonism and efficient virus replication in cell culture and in mice. Proc Natl Acad Sci 120: e2302083120. 10.1073/pnas.2302083120 37607224 PMC10468617

[GAD353495KARC3] Ashok Y, Vela-Rodríguez C, Yang C, Alanen HI, Liu F, Paschal BM, Lehtiö L. 2022. Reconstitution of the DTX3L–PARP9 complex reveals determinants for high-affinity heterodimerization and multimeric assembly. Biochem J 479: 289–304. 10.1042/BCJ20210722 35037691

[GAD353495KARC4] Atasheva S, Akhrymuk M, Frolova EI, Frolov I. 2012. New PARP gene with an anti-alphavirus function. J Virol 86: 8147–8160. 10.1128/JVI.00733-12 22623789 PMC3421642

[GAD353495KARC5] Bachmann SB, Frommel SC, Camicia R, Winkler HC, Santoro R, Hassa PO. 2014. DTX3L and ARTD9 inhibit IRF1 expression and mediate in cooperation with ARTD8 survival and proliferation of metastatic prostate cancer cells. Mol Cancer 13: 125. 10.1186/1476-4598-13-125 24886089 PMC4070648

[GAD353495KARC6] Barbarulo A, Iansante V, Chaidos A, Naresh K, Rahemtulla A, Franzoso G, Karadimitris A, Haskard DO, Papa S, Bubici C. 2013. Poly(ADP-ribose) polymerase family member 14 (PARP14) is a novel effector of the JNK2-dependent pro-survival signal in multiple myeloma. Oncogene 32: 4231–4242. 10.1038/onc.2012.448 23045269

[GAD353495KARC7] Beck C, Robert I, Reina-San-Martin B, Schreiber V, Dantzer F. 2014. Poly(ADP-ribose) polymerases in double-strand break repair: focus on PARP1, PARP2 and PARP3. Exp Cell Res 329: 18–25. 10.1016/j.yexcr.2014.07.003 25017100

[GAD353495KARC8] Bejan DS, Lacoursiere RE, Pruneda JN, Cohen MS. 2025. Ubiquitin is directly linked via an ester to protein-conjugated mono-ADP-ribose. EMBO J 44: 2211–2231. 10.1038/s44318-025-00391-7 40000907 PMC12000418

[GAD353495KARC9] Biaesch K, Knapp S, Korn P. 2023. IFN-induced PARPs-sensors of foreign nucleic acids? Pathogens 12: 457. 10.3390/pathogens12030457 36986379 PMC10057411

[GAD353495KARC10] Boehi F, Manetsch P, Hottiger MO. 2021. Interplay between ADP-ribosyltransferases and essential cell signaling pathways controls cellular responses. Cell Discov 7: 104. 10.1038/s41421-021-00323-9 34725336 PMC8560908

[GAD353495KARC11] Bonfiglio JJ, Fontana P, Zhang Q, Colby T, Gibbs-Seymour I, Atanassov I, Bartlett E, Zaja R, Ahel I, Matic I. 2017. Serine ADP-ribosylation depends on HPF1. Mol Cell 65: 932–940.e6. 10.1016/j.molcel.2017.01.003 28190768 PMC5344681

[GAD353495KARC12] Brady PN, Goel A, Johnson MA. 2019. Poly(ADP-ribose) polymerases in host-pathogen interactions, inflammation, and immunity. Microbiol Mol Biol Rev 83: e00010-19. 10.1128/MMBR.00010-19 30567936 PMC6383445

[GAD353495KARC13] Brooks DM, Anand S, Cohen MS. 2023. Immunomodulatory roles of PARPs: shaping the tumor microenvironment, one ADP-ribose at a time. Curr Opin Chem Biol 77: 102402. 10.1016/j.cbpa.2023.102402 37801755 PMC11975434

[GAD353495KARC14] Buch-Larsen SC, Hendriks IA, Tashiro K, Elsborg JD, Vakhrushev SY, Olsen JV, Lüscher B, Liszczak G, Ahel I, Nielsen ML. 2025. Deciphering cytokine-driven ADP-ribosylation signaling networks via Af1521-based mass spectrometry analysis of labile Glu/Asp-linkages. bioRxiv 10.1101/2025.11.03.686237 PMC13388985 42203779

[GAD353495KARC15] Caprara G, Prosperini E, Piccolo V, Sigismondo G, Melacarne A, Cuomo A, Boothby M, Rescigno M, Bonaldi T, Natoli G. 2018. PARP14 controls the nuclear accumulation of a subset of type I IFN-inducible proteins. J Immunol 200: 2439–2454. 10.4049/jimmunol.1701117 29500242

[GAD353495KARC16] Carter-O'Connell I, Vermehren-Schmaedick A, Jin H, Morgan RK, David LL, Cohen MS. 2018. Combining chemical genetics with proximity-dependent labeling reveals cellular targets of poly(ADP-ribose) polymerase 14 (PARP14). ACS Chem Biol 13: 2841–2848. 10.1021/acschembio.8b00567 30247868

[GAD353495KARC17] Challa S, Nandu T, Kim HB, Gong X, Renshaw CW, Li WC, Tan X, Aljardali MW, Camacho CV, Chen J, 2025. RACK1 MARylation regulates translation and stress granules in ovarian cancer cells. J Cell Biol 224: e202401101. 10.1083/jcb.202401101 39760726 PMC11702359

[GAD353495KARC18] Chambon P, Weill JD, Mandel P. 1963. Nicotinamide mononucleotide activation of new DNA-dependent polyadenylic acid synthesizing nuclear enzyme. Biochem Biophys Res Commun 11: 39–43. 10.1016/0006-291x(63)90024-x 14019961

[GAD353495KARC19] Chatrin C, Zhu K, Ahel I. 2025. The rise of ADP-ribose–ubiquitin. Nat Struct Mol Biol 32: 1582–1585. 10.1038/s41594-025-01651-0 40921856

[GAD353495KARC20] Chatzicharalampous C, Schüler H. 2024. A multidomain PARP14 construct suitable for bacterial expression. Protein Expr Purif 224: 106580. 10.1016/j.pep.2024.106580 39154924

[GAD353495KARC21] Cho SH, Ahn AK, Bhargava P, Lee CH, Eischen CM, McGuinness O, Boothby M. 2011. Glycolytic rate and lymphomagenesis depend on PARP14, an ADP ribosyltransferase of the B aggressive lymphoma (BAL) family. Proc Natl Acad Sci 108: 15972–7. 10.1073/pnas.1017082108 21911376 PMC3179111

[GAD353495KARC22] Correy GJ, Rachman MM, Togo T, Gahbauer S, Doruk YU, Stevens MGV, Jaishankar P, Kelley B, Goldman B, Schmidt M, 2025. Exploration of structure–activity relationships for the SARS-CoV-2 macrodomain from shape-based fragment linking and active learning. Sci Adv 11: eads7187. 10.1126/sciadv.ads7187 40435250 PMC12118597

[GAD353495KARC23] Daugherty MD, Young JM, Kerns JA, Malik HS. 2014. Rapid evolution of PARP genes suggests a broad role for ADP-ribosylation in host-virus conflicts. PLoS Genet 10: e1004403. 10.1371/journal.pgen.1004403 24875882 PMC4038475

[GAD353495KARC24] Delgado-Rodriguez SE, Ryan AP, Daugherty MD. 2023. Recurrent loss of macrodomain activity in host immunity and viral proteins. Pathogens 12: 674. 10.3390/pathogens12050674 37242344 PMC10221186

[GAD353495KARC25] Dhoonmoon A, Nicolae CM. 2023. Mono-ADP-ribosylation by PARP10 and PARP14 in genome stability. NAR Cancer 5: zcad009. 10.1093/narcan/zcad009 36814782 PMC9940457

[GAD353495KARC26] Dhoonmoon A, Schleicher EM, Clements KE, Nicolae CM, Moldovan GL. 2020. Genome-wide CRISPR synthetic lethality screen identifies a role for the ADP-ribosyltransferase PARP14 in DNA replication dynamics controlled by ATR. Nucleic Acids Res 48: 7252–7264. 10.1093/nar/gkaa508 32542389 PMC7367200

[GAD353495KARC27] Dhoonmoon A, Nicolae CM, Moldovan GL. 2022. The KU–PARP14 axis differentially regulates DNA resection at stalled replication forks by MRE11 and EXO1. Nat Commun 13: 5063. 10.1038/s41467-022-32756-5 36030235 PMC9420157

[GAD353495KARC28] Du Z, Fenn S, Tjhen R, James TL. 2008. Structure of a construct of a human poly(C)-binding protein containing the first and second KH domains reveals insights into its regulatory mechanisms. J Biol Chem 283: 28757–28766. 10.1074/jbc.M803046200 18701464 PMC2568903

[GAD353495KARC29] Đukić N, Strømland Ø, Elsborg JD, Munnur D, Zhu K, Schuller M, Chatrin C, Kar P, Duma L, Suyari O, 2023. PARP14 is a PARP with both ADP-ribosyl transferase and hydrolase activities. Sci Adv 9: eadi2687. 10.1126/sciadv.adi2687 37703374 PMC10499325

[GAD353495KARC30] Ebenwaldner C, García Saura AG, Ekström S, Bernfur K, Moche M, Logan DT, Cohen MS, Schüler H. 2025. Regulation of ADP-ribosyl transferase activity by ART domain dimerization in PARP15. Nat Commun 16: 9567. 10.1038/s41467-025-65315-9 41162413 PMC12572374

[GAD353495KARC31] Eddie AM, Chen KW, Schenkel LB, Swinger KK, Molina JR, Kunii K, Raybuck AL, Keilhack H, Gibson-Corley KN, Niepel M, 2022. Selective pharmaceutical inhibition of PARP14 mitigates allergen-induced IgE and mucus overproduction in a mouse model of pulmonary allergic response. Immunohorizons 6: 432–446. 10.4049/immunohorizons.2100107 35817532 PMC10182383

[GAD353495KARC32] Eisemann T, Pascal JM. 2020. Poly(ADP-ribose) polymerase enzymes and the maintenance of genome integrity. Cell Mol Life Sci 77: 19–33. 10.1007/s00018-019-03366-0 31754726 PMC11104942

[GAD353495KARC33] Erdal E, Haider S, Rehwinkel J, Harris AL, McHugh PJ. 2017. A prosurvival DNA damage-induced cytoplasmic interferon response is mediated by end resection factors and is limited by Trex1. Genes Dev 31: 353–369. 10.1101/gad.289769.116 28279982 PMC5358756

[GAD353495KARC34] Fehr AR, Channappanavar R, Jankevicius G, Fett C, Zhao J, Athmer J, Meyerholz DK, Ahel I, Perlman S. 2016. The conserved coronavirus macrodomain promotes virulence and suppresses the innate immune response during severe acute respiratory syndrome coronavirus infection. mBio 7: e01721-16. 10.1128/mBio.01721-16 27965448 PMC5156301

[GAD353495KARC35] Fehr AR, Jankevicius G, Ahel I, Perlman S. 2018. Viral macrodomains: unique mediators of viral replication and pathogenesis. Trends Microbiol 26: 598–610. 10.1016/j.tim.2017.11.011 29268982 PMC6003825

[GAD353495KARC36] Fehr AR, Singh SA, Kerr CM, Mukai S, Higashi H, Aikawa M. 2020. The impact of PARPs and ADP-ribosylation on inflammation and host-pathogen interactions. Genes Dev 34: 341–359. 10.1101/gad.334425.119 32029454 PMC7050484

[GAD353495KARC37] Ficarelli M, Wilson H, Pedro Galão R, Mazzon M, Antzin-Anduetza I, Marsh M, Neil SJ, Swanson CM. 2019. KHNYN is essential for the zinc finger antiviral protein (ZAP) to restrict HIV-1 containing clustered CpG dinucleotides. eLife 8: e46767. 10.7554/eLife.46767 31284899 PMC6615859

[GAD353495KARC38] Fontana P, Bonfiglio JJ, Palazzo L, Bartlett E, Matic I, Ahel I. 2017. Serine ADP-ribosylation reversal by the hydrolase ARH3. eLife 6: e28533. 10.7554/eLife.28533 28650317 PMC5552275

[GAD353495KARC39] Forst AH, Karlberg T, Herzog N, Thorsell AG, Gross A, Feijs KL, Verheugd P, Kursula P, Nijmeijer B, Kremmer E, 2013. Recognition of mono-ADP-ribosylated ARTD10 substrates by ARTD8 macrodomains. Structure 21: 462–475. 10.1016/j.str.2012.12.019 23473667

[GAD353495KARC40] Gahbauer S, Correy GJ, Schuller M, Ferla MP, Doruk YU, Rachman M, Wu T, Diolaiti M, Wang S, Neitz RJ, 2023. Iterative computational design and crystallographic screening identifies potent inhibitors targeting the Nsp3 macrodomain of SARS-CoV-2. Proc Natl Acad Sci 120: e2212931120. 10.1073/pnas.2212931120 36598939 PMC9926234

[GAD353495KARC41] Gao G, Guo X, Goff SP. 2002. Inhibition of retroviral RNA production by ZAP, a CCCH-type zinc finger protein. Science 297: 1703–1706. 10.1126/science.1074276 12215647

[GAD353495KARC42] Gibson BA, Conrad LB, Huang D, Kraus WL. 2017. Generation and characterization of recombinant antibody-like ADP-ribose binding proteins. Biochemistry 56: 6305–6316. 10.1021/acs.biochem.7b00670 29053245 PMC6465537

[GAD353495KARC43] Gorelik A, Paulo JA, Schroeter CB, Lad M, Shurr A, Mastrokalou C, Siddiqi S, Suyari O, Brognard J, Walter D, 2025. CRISPR screens and quantitative proteomics reveal remodeling of the aryl hydrocarbon receptor-driven proteome through PARP7 activity. Proc Natl Acad Sci 122: e2424985122. 10.1073/pnas.2424985122 40493189 PMC12184497

[GAD353495KARC44] Gossmann TI, Schmid KJ. 2011. Selection-driven divergence after gene duplication in Arabidopsis thaliana. J Mol Evol 73: 153–165. 10.1007/s00239-011-9463-2 21965041

[GAD353495KARC45] Gossmann TI, Ziegler M. 2014. Sequence divergence and diversity suggests ongoing functional diversification of vertebrate NAD metabolism. DNA Repair (Amst) 23: 39–48. 10.1016/j.dnarep.2014.07.005 25084685 PMC4248024

[GAD353495KARC46] Gozgit JM, Vasbinder MM, Abo RP, Kunii K, Kuplast-Barr KG, Gui B, Lu AZ, Molina JR, Minissale E, Swinger KK, 2021. PARP7 negatively regulates the type I interferon response in cancer cells and its inhibition triggers antitumor immunity. Cancer Cell 39: 1214–1226.e10. 10.1016/j.ccell.2021.06.018 34375612

[GAD353495KARC47] Groslambert J, Prokhorova E, Ahel I. 2021. ADP-ribosylation of DNA and RNA. DNA Repair 105: 103144. 10.1016/j.dnarep.2021.103144 34116477 PMC8385414

[GAD353495KARC48] Grunewald ME, Chen Y, Kuny C, Maejima T, Lease R, Ferraris D, Aikawa M, Sullivan CS, Perlman S, Fehr AR. 2019. The coronavirus macrodomain is required to prevent PARP-mediated inhibition of virus replication and enhancement of IFN expression. PLoS Pathog 15: e1007756. 10.1371/journal.ppat.1007756 31095648 PMC6521996

[GAD353495KARC49] Grunewald ME, Shaban MG, Mackin SR, Fehr AR, Perlman S. 2020. Murine coronavirus infection activates the aryl hydrocarbon receptor in an indoleamine 2,3-dioxygenase-independent manner, contributing to cytokine modulation and proviral TCDD-inducible-PARP expression. J Virol 94: e01743-19. 10.1128/JVI.01743-19 31694960 PMC7000979

[GAD353495KARC50] Guo X, Carroll JW, Macdonald MR, Goff SP, Gao G. 2004. The zinc finger antiviral protein directly binds to specific viral mRNAs through the CCCH zinc finger motifs. J Virol 78: 12781–7. 10.1128/JVI.78.23.12781-12787.2004 15542630 PMC525010

[GAD353495KARC51] Guo T, Zuo Y, Qian L, Liu J, Yuan Y, Xu K, Miao Y, Feng Q, Chen X, Jin L, 2019. ADP-ribosyltransferase PARP11 modulates the interferon antiviral response by mono-ADP-ribosylating the ubiquitin E3 ligase β-TrCP. Nat Microbiol 4: 1872–1884. 10.1038/s41564-019-0428-3 30988430

[GAD353495KARC52] Hafner A, Bulyk ML, Jambhekar A, Lahav G. 2019. The multiple mechanisms that regulate p53 activity and cell fate. Nat Rev Mol Cell Biol 20: 199–210. 10.1038/s41580-019-0110-x 30824861

[GAD353495KARC53] Hayakawa S, Shiratori S, Yamato H, Kameyama T, Kitatsuji C, Kashigi F, Goto S, Kameoka S, Fujikura D, Yamada T, 2011. ZAPS is a potent stimulator of signaling mediated by the RNA helicase RIG-I during antiviral responses. Nat Immunol 12: 37–44. 10.1038/ni.1963 21102435

[GAD353495KARC54] Hendriks IA, Buch-Larsen SC, Prokhorova E, Elsborg JD, Rebak AKLFS, Zhu K, Ahel D, Lukas C, Ahel I, Nielsen ML. 2021. The regulatory landscape of the human HPF1- and ARH3-dependent ADP-ribosylome. Nat Commun 12: 5893. 10.1038/s41467-021-26172-4 34625544 PMC8501107

[GAD353495KARC55] Higashi H, Maejima T, Lee LH, Yamazaki Y, Hottiger MO, Singh SA, Aikawa M. 2019. A study into the ADP-ribosylome of IFN-γ-stimulated THP-1 human macrophage-like cells identifies ARTD8/PARP14 and ARTD9/PARP9 ADP-ribosylation. J Proteome Res 18: 1607–1622. 10.1021/acs.jproteome.8b00895 30848916 PMC6456868

[GAD353495KARC56] Huang D, Kraus WL. 2022. The expanding universe of PARP1-mediated molecular and therapeutic mechanisms. Mol Cell 82: 2315–2334. 10.1016/j.molcel.2022.02.021 35271815 PMC9232969

[GAD353495KARC57] Huang D, Camacho CV, Setlem R, Ryu KW, Parameswaran B, Gupta RK, Kraus WL. 2020. Functional interplay between histone H2B ADP-ribosylation and phosphorylation controls adipogenesis. Mol Cell 79: 934–949.e14. 10.1016/j.molcel.2020.08.002 32822587 PMC7502539

[GAD353495KARC58] Iansante V, Choy PM, Fung SW, Liu Y, Chai JG, Dyson J, Del Rio A, D'Santos C, Williams R, Chokshi S, 2015. PARP14 promotes the Warburg effect in hepatocellular carcinoma by inhibiting JNK1-dependent PKM2 phosphorylation and activation. Nat Commun 6: 7882. 10.1038/ncomms8882 26258887 PMC4918319

[GAD353495KARC59] Iqbal MB, Johns M, Cao J, Liu Y, Yu SC, Hyde GD, Laffan MA, Marchese FP, Cho SH, Clark AR, 2014. PARP-14 combines with tristetraprolin in the selective posttranscriptional control of macrophage tissue factor expression. Blood 124: 3646–3655. 10.1182/blood-2014-07-588046 25293769 PMC4256914

[GAD353495KARC60] Iwata H, Goettsch C, Sharma A, Ricchiuto P, Goh WW, Halu A, Yamada I, Yoshida H, Hara T, Wei M, 2016. PARP9 and PARP14 cross-regulate macrophage activation via STAT1 ADP-ribosylation. Nat Commun 7: 12849. 10.1038/ncomms12849 27796300 PMC5095532

[GAD353495KARC61] Jankevicius G, Hassler M, Golia B, Rybin V, Zacharias M, Timinszky G, Ladurner AG. 2013. A family of macrodomain proteins reverses cellular mono-ADP-ribosylation. Nat Struct Mol Biol 20: 508–514. 10.1038/nsmb.2523 23474712 PMC7097781

[GAD353495KARC62] Javed Z, Nguyen HH, Harker KK, Mohr CM, Vano P, Wallace SR, Silvers C, Sim C, Turumella S, Flinn A, 2023. Using TLC-MALDI-TOF to interrogate in vitro peptidyl proximal preferences of PARP14 and glycohydrolase specificity. Molecules 28: 6061. 10.3390/molecules28166061 37630315 PMC10459978

[GAD353495KARC63] Jiang L, Mu H, Xing H, Zhu F, Zhao X, Wu Y. 2025. DTX3L e3 ligase: molecular functions, regulatory mechanisms, and potential as a novel cancer biomarker and therapeutic target. Int J Biol Macromol 321: 146194. 10.1016/j.ijbiomac.2025.146194 40695431

[GAD353495KARC64] Juszczynski P, Kutok JL, Li C, Mitra J, Aguiar RC, Shipp MA. 2006. *BAL1* and *BBAP* are regulated by a γ interferon-responsive bidirectional promoter and are overexpressed in diffuse large B-cell lymphomas with a prominent inflammatory infiltrate. Mol Cell Biol 26: 5348–5359. 10.1128/MCB.02351-05 16809771 PMC1592708

[GAD353495KARC65] Kar P, Chatrin C, Đukić N, Suyari O, Schuller M, Zhu K, Prokhorova E, Bigot N, Baretić D, Ahel J, 2024. PARP14 and PARP9/DTX3L regulate interferon-induced ADP-ribosylation. EMBO J 43: 2929–2953. 10.1038/s44318-024-00126-0 38834853 PMC11251020

[GAD353495KARC66] Kerns JA, Emerman M, Malik HS. 2008. Positive selection and increased antiviral activity associated with the PARP-containing isoform of human zinc-finger antiviral protein. PLoS Genet 4: e21. 10.1371/journal.pgen.0040021 18225958 PMC2213710

[GAD353495KARC67] Kleine H, Poreba E, Lesniewicz K, Hassa PO, Hottiger MO, Litchfield DW, Shilton BH, Lüscher B. 2008. Substrate-assisted catalysis by PARP10 limits its activity to mono-ADP-ribosylation. Mol Cell 32: 57–69. 10.1016/j.molcel.2008.08.009 18851833

[GAD353495KARC68] Kloet MS, Chatrin C, Mukhopadhyay R, van Tol BDM, Smith R, Rotman SA, Tjokrodirijo RTN, Zhu K, Gorelik A, Maginn L, 2025. Identification of RNF114 as ADPr–Ub reader through non-hydrolysable ubiquitinated ADP-ribose. Nat Commun 16: 6319. 10.1038/s41467-025-61111-7 40634336 PMC12241653

[GAD353495KARC69] Krieg S, Pott F, Potthoff L, Verheirstraeten M, Bütepage M, Golzmann A, Lippok B, Goffinet C, Lüscher B, Korn P. 2023. Mono-ADP-ribosylation by PARP10 inhibits Chikungunya virus nsP2 proteolytic activity and viral replication. Cell Mol Life Sci 80: 72. 10.1007/s00018-023-04717-8 36840772 PMC9959937

[GAD353495KARC70] Kumar S, Hedges SB. 1998. A molecular timescale for vertebrate evolution. Nature 392: 917–920. 10.1038/31927 9582070

[GAD353495KARC71] Lacoursiere RE, Upadhyaya K, Kaur Sidhu J, Rodriguez Siordia I, Bejan DS, Cohen MS, Pruneda JN. 2025. RNF114 and RNF166 exemplify reader-writer E3 ligases that extend K11 polyubiquitin onto sites of MARUbylation. EMBO J 44: 5993–6018. 10.1038/s44318-025-00577-z 41039157 PMC12583694

[GAD353495KARC72] Langelier MF, Planck JL, Roy S, Pascal JM. 2012. Structural basis for DNA damage-dependent poly(ADP-ribosyl)ation by human PARP-1. Science 336: 728–732. 10.1126/science.1216338 22582261 PMC3532513

[GAD353495KARC73] Leshem R, Sefton KN, Wong CW, Lin IH, Isaac DT, Niepel M, Hurlstone A. 2025. Combined PARP14 inhibition and PD-1 blockade promotes cytotoxic T cell quiescence and modulates macrophage polarization in relapsed melanoma. J Immunother Cancer 13: e010683. 10.1136/jitc-2024-010683 39870492 PMC11772928

[GAD353495KARC74] Leung AKL, Griffin DE, Bosch J, Fehr AR. 2022. The conserved macrodomain is a potential therapeutic target for coronaviruses and alphaviruses. Pathogens 11: 94. 10.3390/pathogens11010094 35056042 PMC8780475

[GAD353495KARC75] Li X, Song Y. 2024. Targeting SARS-CoV-2 nonstructural protein 3: function, structure, inhibition, and perspective in drug discovery. Drug Discov Today 29: 103832. 10.1016/j.drudis.2023.103832 37977285 PMC10872262

[GAD353495KARC76] Li C, Debing Y, Jankevicius G, Neyts J, Ahel I, Coutard B, Canard B. 2016. Viral macro domains reverse protein ADP-ribosylation. J Virol 90: 8478–8486. 10.1128/JVI.00705-16 27440879 PMC5021415

[GAD353495KARC77] Li L, Zhao H, Liu P, Li C, Quanquin N, Ji X, Sun N, Du P, Qin CF, Lu N, 2018. PARP12 suppresses Zika virus infection through PARP-dependent degradation of NS1 and NS3 viral proteins. Sci Signal 11: eaas9332. 10.1126/scisignal.aas9332 29921658 PMC6434931

[GAD353495KARC78] Li L, Shi Y, Li S, Liu J, Zu S, Xu X, Gao M, Sun N, Pan C, Peng L, 2021. ADP-ribosyltransferase PARP11 suppresses Zika virus in synergy with PARP12. Cell Biosci 11: 116. 10.1186/s13578-021-00628-y 34187568 PMC8240438

[GAD353495KARC79] Lüscher B, Ahel I, Altmeyer M, Ashworth A, Bai P, Chang P, Cohen M, Corda D, Dantzer F, Daugherty MD, 2022. ADP-ribosyltransferases, an update on function and nomenclature. FEBS J 289: 7399–7410. 10.1111/febs.16142 34323016 PMC9027952

[GAD353495KARC80] Maréchal A, Li JM, Ji XY, Wu CS, Yazinski SA, Nguyen HD, Liu S, Jiménez AE, Jin J, Zou L. 2014. PRP19 transforms into a sensor of RPA–ssDNA after DNA damage and drives ATR activation via a ubiquitin-mediated circuitry. Mol Cell 53: 235–246. 10.1016/j.molcel.2013.11.002 24332808 PMC3946837

[GAD353495KARC81] Maris C, Dominguez C, Allain FH. 2005. The RNA recognition motif, a plastic RNA-binding platform to regulate post-transcriptional gene expression. FEBS J 272: 2118–2131. 10.1111/j.1742-4658.2005.04653.x 15853797

[GAD353495KARC82] Mashimo M, Shimizu A, Mori A, Hamaguchi A, Fukushima K, Seira N, Fujii T, Fujino H. 2022. PARP14 regulates EP4 receptor expression in human colon cancer HCA-7 cells. Biochem Biophys Res Commun 623: 133–139. 10.1016/j.bbrc.2022.07.055 35914351

[GAD353495KARC83] Meagher JL, Takata M, Gonçalves-Carneiro D, Keane SC, Rebendenne A, Ong H, Orr VK, MacDonald MR, Stuckey JA, Bieniasz PD, 2019. Structure of the zinc-finger antiviral protein in complex with RNA reveals a mechanism for selective targeting of CG-rich viral sequences. Proc Natl Acad Sci 116: 24303–24309. 10.1073/pnas.1913232116 31719195 PMC6883784

[GAD353495KARC84] Mehrotra P, Riley JP, Patel R, Li F, Voss L, Goenka S. 2011. PARP-14 functions as a transcriptional switch for Stat6-dependent gene activation. J Biol Chem 286: 1767–1776. 10.1074/jbc.M110.157768 21081493 PMC3023471

[GAD353495KARC85] Mehrotra P, Hollenbeck A, Riley JP, Li F, Patel RJ, Akhtar N, Goenka S. 2013. Poly(ADP-ribose) polymerase 14 and its enzyme activity regulates T(H)2 differentiation and allergic airway disease. J Allergy Clin Immunol 131: 521–531.e1-12. 10.1016/j.jaci.2012.06.015 22841009 PMC3502685

[GAD353495KARC86] Mehrotra P, Krishnamurthy P, Sun J, Goenka S, Kaplan MH. 2015. Poly-ADP-ribosyl polymerase-14 promotes T helper 17 and follicular T helper development. Immunology 146: 537–546. 10.1111/imm.12515 26222149 PMC4693893

[GAD353495KARC87] Mikolčević P, Hloušek-Kasun A, Ahel I, Mikoč A. 2021. ADP-ribosylation systems in bacteria and viruses. Comput Struct Biotechnol J 19: 2366–2383. 10.1016/j.csbj.2021.04.023 34025930 PMC8120803

[GAD353495KARC88] Mo W, Zhang F, Wang C, Ding X, Ren L. 2025. PARP14-mediated glycolysis enhances tamoxifen resistance in estrogen receptor + breast cancer cells. Discov Oncol 16: 1135. 10.1007/s12672-025-02404-7 40526225 PMC12174036

[GAD353495KARC89] Munnur D, Ahel I. 2017. Reversible mono-ADP-ribosylation of DNA breaks. FEBS J 284: 4002–4016. 10.1111/febs.14297 29054115 PMC5725667

[GAD353495KARC90] Munnur D, Bartlett E, Mikolčević P, Kirby IT, Rack JGM, Mikoč A, Cohen MS, Ahel I. 2019. Reversible ADP-ribosylation of RNA. Nucleic Acids Res 47: 5658–5669. 10.1093/nar/gkz305 31216043 PMC6582358

[GAD353495KARC91] Musheev MU, Schomacher L, Basu A, Han D, Krebs L, Scholz C, Niehrs C. 2022. Mammalian N1-adenosine PARylation is a reversible DNA modification. Nat Commun 13: 6138. 10.1038/s41467-022-33731-w 36253381 PMC9576699

[GAD353495KARC92] Nicolae CM, Aho ER, Choe KN, Constantin D, Hu HJ, Lee D, Myung K, Moldovan GL. 2015. A novel role for the mono-ADP-ribosyltransferase PARP14/ARTD8 in promoting homologous recombination and protecting against replication stress. Nucleic Acids Res 43: 3143–3153. 10.1093/nar/gkv147 25753673 PMC4381061

[GAD353495KARC93] O'Connor MJ, Thakar T, Nicolae CM, Moldovan GL. 2021. PARP14 regulates cyclin D1 expression to promote cell-cycle progression. Oncogene 40: 4872–4883. 10.1038/s41388-021-01881-8 34158578 PMC8384455

[GAD353495KARC94] O'Connor JJ, Ferraris D, Fehr AR. 2023. An update on the current state of SARS-CoV-2 Mac1 inhibitors. Pathogens 12: 1221. 10.3390/pathogens12101221 37887737 PMC10610136

[GAD353495KARC95] Oppenheimer NJ, Bodley JW. 1981. Diphtheria toxin site and configuration of ADP-ribosylation of diphthamide in elongation factor 2. J Biol Chem 256: 8579–8581. 10.1016/S0021-9258(19)68883-6 6267047

[GAD353495KARC96] Palazzo L, Ahel I. 2018. PARPs in genome stability and signal transduction: implications for cancer therapy. Biochem Soc Trans 46: 1681–1695. 10.1042/BST20180418 30420415 PMC6299239

[GAD353495KARC97] Palazzo L, Leidecker O, Prokhorova E, Dauben H, Matic I, Ahel I. 2018. Serine is the major residue for ADP-ribosylation upon DNA damage. eLife 7: e34334. 10.7554/eLife.34334 29480802 PMC5837557

[GAD353495KARC98] Palazzo L, Suskiewicz MJ, Ahel I. 2021. Serine ADP-ribosylation in DNA-damage response regulation. Curr Opin Genet Dev 71: 106–113. 10.1016/j.gde.2021.07.005 34340015

[GAD353495KARC99] Parthasarathy S, Fehr AR. 2022. PARP14: a key ADP-ribosylating protein in host–virus interactions? PLoS Pathog 18: e1010535. 10.1371/journal.ppat.1010535 35679255 PMC9182250

[GAD353495KARC100] Parthasarathy S, Saenjamsai P, Hao H, Ferkul A, Pfannenstiel JJ, Bejan DS, Chen Y, Suder EL, Schwarting N, Aikawa M, 2025. PARP14 is an interferon-induced host factor that promotes IFN production and affects the replication of multiple viruses. mBio 16: e0229925. 10.1128/mbio.02299-25 40937852 PMC12505956

[GAD353495KARC101] Perina D, Mikoč A, Ahel J, Ćetković H, Žaja R, Ahel I. 2014. Distribution of protein poly(ADP-ribosyl)ation systems across all domains of life. DNA Repair (Amst) 23: 4–16. 10.1016/j.dnarep.2014.05.003 24865146 PMC4245714

[GAD353495KARC102] Perrard J, Gao K, Ring K, Smith S. 2025. Deltex and RING-UIM E3 ligases cooperate to create a ubiquitin-ADP-ribose hybrid mark on tankyrase, promoting its stabilization. Sci Adv 11: eadx7172. 10.1126/sciadv.adx7172 40901936 PMC12407064

[GAD353495KARC103] Pfannenstiel JJ, Duong MTH, Cluff D, Sherrill LM, Colquhoun I, Cadoux G, Thorne D, Pääkkönen J, Schemmel NF, O'Connor J, 2025. Identification of a series of pyrrolo-pyrimidine-based SARS-CoV-2 Mac1 inhibitors that repress coronavirus replication. mBio 16: e0386524. 10.1128/mbio.03865-24 40407321 PMC12153294

[GAD353495KARC104] Polasek TM, Cole A, Bozón V, Manyak E, Novak J, Yang B, Johnston BA, Parasuraman S, Paneliya KJ, Schuck V. 2025. First-in-human phase 1 study to evaluate the clinical pharmacology properties of RBN-3143, a novel inhibitor of mono-adenosine diphosphate ribosyltransferase-PARP14. Clin Pharmacol Drug Dev 14: 493–504. 10.1002/cpdd.1539 40304706 PMC12209991

[GAD353495KARC105] Prokarenkaite R, Kuodyte K, Gudoityte G, Budginaite E, Naumovas D, Strainiene E, Velickevicius K, Dulskas A, Sileika E, Venius J, 2025. PARP9–PARP13–PARP14 axis tunes colorectal cancer response to radiotherapy. J Exp Clin Cancer Res 44: 199. 10.1186/s13046-025-03439-y 40646573 PMC12247367

[GAD353495KARC106] Prokhorova E, Zobel F, Smith R, Zentout S, Gibbs-Seymour I, Schützenhofer K, Peters A, Groslambert J, Zorzini V, Agnew T, 2021. Serine-linked PARP1 auto-modification controls PARP inhibitor response. Nat Commun 12: 4055. 10.1038/s41467-021-24361-9 34210965 PMC8249464

[GAD353495KARC107] Rack JG, Perina D, Ahel I. 2016. Macrodomains: structure, function, evolution, and catalytic activities. Annu Rev Biochem 85: 431–454. 10.1146/annurev-biochem-060815-014935 26844395

[GAD353495KARC108] Rack JGM, Palazzo L, Ahel I. 2020a. (ADP-ribosyl) hydrolases: structure, function, and biology. Genes Dev 34: 263–284. 10.1101/gad.334631.119 32029451 PMC7050489

[GAD353495KARC109] Rack JGM, Zorzini V, Zhu Z, Schuller M, Ahel D, Ahel I. 2020b. Viral macrodomains: a structural and evolutionary assessment of the pharmacological potential. Open Biol 10: 200237. 10.1098/rsob.200237 33202171 PMC7729036

[GAD353495KARC110] Raja R, Biswas B, Abraham R, Wang Y, Chang CY, Hendriks IA, Buch-Larsen SC, Liu H, Yang X, Wang C, 2025. Interferon-induced PARP14-mediated ADP-ribosylation in p62 bodies requires the ubiquitin-proteasome system. EMBO J 44: 2741–2773. 10.1038/s44318-025-00421-4 40195501 PMC12084362

[GAD353495KARC111] Ribeiro VC, Russo LC, Hoch NC. 2024. PARP14 is regulated by the PARP9/DTX3L complex and promotes interferon γ-induced ADP-ribosylation. EMBO J 43: 2908–2928. 10.1038/s44318-024-00125-1 38834852 PMC11251048

[GAD353495KARC112] Ribeiro VC, Russo LC, González Duré DM, Hoch NC. 2025. Interferon-induced ADP-ribosylation: technical developments driving ICAB discovery. Biosci Rep 45: BSR20240986. 10.1042/BSR20240986 40014063 PMC12096948

[GAD353495KARC113] Rijpkema KJ, Schuller M, van der Veer MS, Rieken S, Chang DLR, Balić P, Todorov A, Minnee H, Wijngaarden S, Matos IA, 2024. Synthesis of structural ADP-ribose analogues as inhibitors for SARS-CoV-2 macrodomain 1. Org Lett 26: 5700–5704. 10.1021/acs.orglett.4c01792 38935522 PMC11249776

[GAD353495KARC114] Rosenthal F, Feijs KL, Frugier E, Bonalli M, Forst AH, Imhof R, Winkler HC, Fischer D, Caflisch A, Hassa PO, 2013. Macrodomain-containing proteins are new mono-ADP-ribosylhydrolases. Nat Struct Mol Biol 20: 502–507. 10.1038/nsmb.2521 23474714

[GAD353495KARC115] Roy A, Alhammad YM, McDonald P, Johnson DK, Zhuo J, Wazir S, Ferraris D, Lehtiö L, Leung AKL, Fehr AR. 2022. Discovery of compounds that inhibit SARS-CoV-2 Mac1-ADP-ribose binding by high-throughput screening. Antiviral Res 203: 105344. 10.1016/j.antiviral.2022.105344 35598780 PMC9119168

[GAD353495KARC116] Saleh H, Liloglou T, Rigden DJ, Parsons JL, Grundy GJ. 2024. KH-like domains in PARP9/DTX3L and PARP14 coordinate protein-protein interactions to promote cancer cell survival. J Mol Biol 436: 168434. 10.1016/j.jmb.2023.168434 38182103 PMC11080071

[GAD353495KARC117] Sanderson DJ, Rodriguez KM, Bejan DS, Olafsen NE, Bohn ID, Kojic A, Sundalam S, Siordia IR, Duell AK, Deng N, 2023. Structurally distinct PARP7 inhibitors provide new insights into the function of PARP7 in regulating nucleic acid-sensing and IFN-β signaling. Cell Chem Biol 30: 43–54.e8. 10.1016/j.chembiol.2022.11.012 36529140 PMC9868104

[GAD353495KARC118] Santinelli-Pestana DV, Aikawa E, Singh SA, Aikawa M. 2023. PARPs and ADP-ribosylation in chronic inflammation: a focus on macrophages. Pathogens 12: 964. 10.3390/pathogens12070964 37513811 PMC10386340

[GAD353495KARC119] Schenkel LB, Molina JR, Swinger KK, Abo R, Blackwell DJ, Lu AZ, Cheung AE, Church WD, Kunii K, Kuplast-Barr KG, 2021. A potent and selective PARP14 inhibitor decreases protumor macrophage gene expression and elicits inflammatory responses in tumor explants. Cell Chem Biol 28: 1158–1168.e13. 10.1016/j.chembiol.2021.02.010 33705687

[GAD353495KARC120] Schüler M, Riedel K, Gibbs-Seymour I, Uth K, Sieg C, Gehring AP, Ahel I, Bracher F, Kessler BM, Elkins JM, 2017. Discovery of a selective allosteric inhibitor targeting macrodomain 2 of polyadenosine-diphosphate-ribose polymerase 14. ACS Chem Biol 12: 2866–2874. 10.1021/acschembio.7b00445 28991428 PMC6089342

[GAD353495KARC121] Schuller M, Butler RE, Ariza A, Tromans-Coia C, Jankevicius G, Claridge TDW, Kendall SL, Goh S, Stewart GR, Ahel I. 2021a. Molecular basis for DarT ADP-ribosylation of a DNA base. Nature 596: 597–602. 10.1038/s41586-021-03825-4 34408320

[GAD353495KARC122] Schuller M, Correy GJ, Gahbauer S, Fearon D, Wu T, Díaz RE, Young ID, Carvalho Martins L, Smith DH, Schulze-Gahmen U, 2021b. Fragment binding to the Nsp3 macrodomain of SARS-CoV-2 identified through crystallographic screening and computational docking. Sci Adv 7: eabf8711. 10.1126/sciadv.abf8711 33853786 PMC8046379

[GAD353495KARC123] Schuller M, Raggiaschi R, Mikolcevic P, Rack JGM, Ariza A, Zhang Y, Ledermann R, Tang C, Mikoc A, Ahel I. 2023. Molecular basis for the reversible ADP-ribosylation of guanosine bases. Mol Cell 83: 2303–2315.e6. 10.1016/j.molcel.2023.06.013 37390817 PMC11543638

[GAD353495KARC124] Sharifi R, Morra R, Appel CD, Tallis M, Chioza B, Jankevicius G, Simpson MA, Matic I, Ozkan E, Golia B, 2013. Deficiency of terminal ADP-ribose protein glycohydrolase TARG1/C6orf130 in neurodegenerative disease. EMBO J 32: 1225–1237. 10.1038/emboj.2013.51 23481255 PMC3642678

[GAD353495KARC125] Suryawanshi RK, Jaishankar P, Correy GJ, Rachman MM, O'Leary PC, Taha TY, Matsui Y, Zapatero-Belinchón FJ, McCavitt-Malvido M, Doruk YU, 2025. The Mac1 ADP-ribosylhydrolase is a therapeutic target for SARS-CoV2. eLife 14: RP103484. 10.7554/eLife.103484 41258893 PMC12629595

[GAD353495KARC126] Suskiewicz MJ, Zobel F, Ogden TEH, Fontana P, Ariza A, Yang JC, Zhu K, Bracken L, Hawthorne WJ, Ahel D, 2020. HPF1 completes the PARP active site for DNA damage-induced ADP-ribosylation. Nature 579: 598–602. 10.1038/s41586-020-2013-6 32028527 PMC7104379

[GAD353495KARC127] Suskiewicz MJ, Munnur D, Strømland Ø, Yang JC, Easton LE, Chatrin C, Zhu K, Baretić D, Goffinont S, Schuller M, 2023a. Updated protein domain annotation of the PARP protein family sheds new light on biological function. Nucleic Acids Res 51: 8217–8236. 10.1093/nar/gkad514 37326024 PMC10450202

[GAD353495KARC128] Suskiewicz MJ, Prokhorova E, Rack JGM, Ahel I. 2023b. ADP-ribosylation from molecular mechanisms to therapeutic implications. Cell 186: 4475–4495. 10.1016/j.cell.2023.08.030 37832523 PMC10789625

[GAD353495KARC129] Takeyama K, Aguiar RC, Gu L, He C, Freeman GJ, Kutok JL, Aster JC, Shipp MA. 2003. The BAL-binding protein BBAP and related Deltex family members exhibit ubiquitin-protein isopeptide ligase activity. J Biol Chem 278: 21930–7. 10.1074/jbc.M301157200 12670957

[GAD353495KARC130] Talhaoui I, Lebedeva NA, Zarkovic G, Saint-Pierre C, Kutuzov MM, Sukhanova MV, Matkarimov BT, Gasparutto D, Saparbaev MK, Lavrik OI, 2016. Poly(ADP-ribose) polymerases covalently modify strand break termini in DNA fragments in vitro. Nucleic Acids Res 44: 9279–9295. 10.1093/nar/gkw675 27471034 PMC5100588

[GAD353495KARC131] Tang L, Lu Y, van der Heijden FLAM, Chen Y, Jiang S, Meeuwenoord NJ, Liu L, Yu Z, Chen Z, Schuller M, 2026. Chemical synthesis of native ADP-ribosylated oligonucleotides enables analysis of DNA ADP-ribosylation hydrolase specificity. J Am Chem Soc 148: 4270–4282. 10.1021/jacs.5c17532 41505235 PMC12879935

[GAD353495KARC132] Tashiro K, Wijngaarden S, Mohapatra J, Rack JGM, Ahel I, Filippov DV, Liszczak G. 2023. Chemoenzymatic and synthetic approaches to investigate aspartate- and glutamate-ADP-ribosylation. J Am Chem Soc 145: 14000–14009. 10.1021/jacs.3c03771 37315125 PMC11065122

[GAD353495KARC133] Teloni F, Altmeyer M. 2016. Readers of poly(ADP-ribose): designed to be fit for purpose. Nucleic Acids Res 44: 993–1006. 10.1093/nar/gkv1383 26673700 PMC4756826

[GAD353495KARC134] Torretta A, Chatzicharalampous C, Ebenwaldner C, Schüler H. 2023. PARP14 is a writer, reader, and eraser of mono-ADP-ribosylation. J Biol Chem 299: 105096. 10.1016/j.jbc.2023.105096 37507011 PMC10470015

[GAD353495KARC135] Valverde R, Edwards L, Regan L. 2008. Structure and function of KH domains. FEBS J 275: 2712–2726. 10.1111/j.1742-4658.2008.06411.x 18422648

[GAD353495KARC136] Vedantham M, Polari L, Poosakkannu A, Pinto RG, Sakari M, Laine J, Sipilä P, Määttä J, Gerke H, Rissanen T, 2024. Body-wide genetic deficiency of poly(ADP-ribose) polymerase 14 sensitizes mice to colitis. FASEB J 38: e23775. 10.1096/fj.202400484R38967223

[GAD353495KARC137] Vyas S, Matic I, Uchima L, Rood J, Zaja R, Hay RT, Ahel I, Chang P. 2014. Family-wide analysis of poly(ADP-ribose) polymerase activity. Nat Commun 5: 4426. 10.1038/ncomms5426 25043379 PMC4123609

[GAD353495KARC138] Wahlberg E, Karlberg T, Kouznetsova E, Markova N, Macchiarulo A, Thorsell AG, Pol E, Frostell A, Ekblad T, Oncu D, 2012. Family-wide chemical profiling and structural analysis of PARP and tankyrase inhibitors. Nat Biotechnol 30: 283–288. 10.1038/nbt.2121 22343925

[GAD353495KARC139] Wallace SR, Chihab LY, Yamasaki M, Yoshinaga BT, Torres YM, Rideaux D, Javed Z, Turumella S, Zhang M, Lawton DR, 2021. Rapid analysis of ADP-ribosylation dynamics and site-specificity using TLC-MALDI. ACS Chem Biol 16: 2137–2143. 10.1021/acschembio.1c00542 34647721 PMC8609518

[GAD353495KARC140] Wang S, Huang J, Zeng T, Chen Y, Xu Y, Zhang B. 2025. Parps in immune response: potential targets for cancer immunotherapy. Biochem Pharmacol 234: 116803. 10.1016/j.bcp.2025.116803 39965743

[GAD353495KARC141] Wierbiłowicz K, Yang CS, Almaghasilah A, Wesołowski PA, Pracht P, Dworak NM, Masur J, Wijngaarden S, Filippov DV, Wales DJ, 2025. Parp7 generates an ADP-ribosyl degron that controls negative feedback of androgen signaling. EMBO J 44: 4720–4744. 10.1038/s44318-025-00510-4 40681873 PMC12402299

[GAD353495KARC142] Wondisford AR, Lee J, Lu R, Schuller M, Groslambert J, Bhargava R, Schamus-Haynes S, Cespedes LC, Opresko PL, Pickett HA, 2024. Deregulated DNA ADP-ribosylation impairs telomere replication. Nat Struct Mol Biol 31: 791–800. 10.1038/s41594-024-01279-6 38714889 PMC11102865

[GAD353495KARC143] Wong CW, Evangelou C, Sefton KN, Leshem R, Zhang W, Gopalan V, Chattrakarn S, Fernandez Carro ML, Uzuner E, Mole H, 2023. PARP14 inhibition restores PD-1 immune checkpoint inhibitor response following IFNγ-driven acquired resistance in preclinical cancer models. Nat Commun 14: 5983. 10.1038/s41467-023-41737-1 37752135 PMC10522711

[GAD353495KARC144] Wu S, Zeng X, Liu J, Cong K, Lou S, Li Z, Wei P, Shao L, Zhang Y, Qu L, 2025. Discovery and optimization of potent and highly selective PARP14 inhibitors for the treatment of atopic dermatitis. J Med Chem 68: 9755–9776. 10.1021/acs.jmedchem.5c00564 40239060

[GAD353495KARC145] Yan Q, Ding J, Khan SJ, Lawton LN, Shipp MA. 2023. DTX3L e3 ligase targets p53 for degradation at poly ADP-ribose polymerase-associated DNA damage sites. iScience 26: 106444. 10.1016/j.isci.2023.106444 37096048 PMC10122052

[GAD353495KARC146] Yanagawa T, Funasaka T, Tsutsumi S, Hu H, Watanabe H, Raz A. 2007. Regulation of phosphoglucose isomerase/autocrine motility factor activities by the poly(ADP-ribose) polymerase family-14. Cancer Res 67: 8682–8689. 10.1158/0008-5472.CAN-07-1586 17875708

[GAD353495KARC147] Yang CS, Jividen K, Spencer A, Dworak N, Ni L, Oostdyk LT, Chatterjee M, Kuśmider B, Reon B, Parlak M, 2017. Ubiquitin modification by the E3 ligase/ADP-ribosyltransferase Dtx3L/Parp9. Mol Cell 66: 503–516.e5. 10.1016/j.molcel.2017.04.028 28525742 PMC5556935

[GAD353495KARC148] Zhang Y, Mao D, Roswit WT, Jin X, Patel AC, Patel DA, Agapov E, Wang Z, Tidwell RM, Atkinson JJ, 2015. PARP9–DTX3L ubiquitin ligase targets host histone H2BJ and viral 3C protease to enhance interferon signaling and control viral infection. Nat Immunol 16: 1215–1227. 10.1038/ni.3279 26479788 PMC4653074

[GAD353495KARC149] Zhang L, Chen W, Shi Z, Shang Z. 2024. PARP14 correlates with GBM proliferation and poor prognosis by elevating expression of SAMD/SAMD9L. Ir J Med Sci 193: 585–593. 10.1007/s11845-023-03500-9 37612499

[GAD353495KARC150] Zhu K, Suskiewicz MJ, Hloušek-Kasun A, Meudal H, Mikoč A, Aucagne V, Ahel D, Ahel I. 2022a. DELTEX e3 ligases ubiquitylate ADP-ribosyl modification on protein substrates. Sci Adv 8: eadd4253. 10.1126/sciadv.add4253 36197986 PMC7615817

[GAD353495KARC151] Zhu Y, Liu Z, Wan Y, Zou L, Liu L, Ding S, Lu C, Qiu F. 2022b. PARP14 promotes the growth and glycolysis of acute myeloid leukemia cells by regulating HIF-1α expression. Clin Immunol 242: 109094. 10.1016/j.clim.2022.109094 35944879

[GAD353495KARC152] Zhu Z, Weng S, Zheng F, Zhao Q, Xu Y, Wu J. 2023. Identification of poly(ADP-ribose) polymerase 9 (PARP9) as a potent suppressor for *Mycobacterium tuberculosis* infection. Phenomics 4: 158–170. 10.1007/s43657-023-00112-2 38884060 PMC11169154

[GAD353495KARC153] Zhu K, Chatrin C, Suskiewicz MJ, Aucagne V, Foster B, Kessler BM, Gibbs-Seymour I, Ahel D, Ahel I. 2024a. Ubiquitylation of nucleic acids by DELTEX ubiquitin E3 ligase DTX3L. EMBO Rep 25: 4172–4189. 10.1038/s44319-024-00235-1 39242775 PMC11467253

[GAD353495KARC154] Zhu K, Suskiewicz MJ, Chatrin C, Strømland Ø, Dorsey BW, Aucagne V, Ahel D, Ahel I. 2024b. DELTEX e3 ligases ubiquitylate ADP-ribosyl modification on nucleic acids. Nucleic Acids Res 52: 801–815. 10.1093/nar/gkad1119 38000390 PMC10810221

[GAD353495KARC155] Zhu K, Chatrin C, Smith R, Ahel D, Ahel I. 2025. Interplay between ubiquitination and ADP-ribosylation and the case of dual modification ADPr–Ub. Essays Biochem 69: 267–279. 10.1042/EBC20253040 41065402 PMC12687431

